# A calcium-sensing receptor dileucine motif directs internalization to spatially distinct endosomal signaling pathways

**DOI:** 10.1016/j.isci.2025.112651

**Published:** 2025-05-13

**Authors:** Rachael A. Wyatt, Meurig T. Gallagher, Ling Zha, Christopher J. McCabe, Caroline M. Gorvin

**Affiliations:** 1 Institute of Metabolism and Systems Research (IMSR) and Centre for Endocrinology, Diabetes and Metabolism (CEDAM), Birmingham Health Partners, University of Birmingham, Birmingham, UK; 2 Centre of Membrane Proteins and Receptors (COMPARE), Universities of Birmingham & Nottingham, Birmingham, UK; 3 Centre for Systems Modelling and Quantitative Biomedicine, University of Birmingham, Birmingham, UK

**Keywords:** Molecular biology, Cell biology

## Abstract

G protein-coupled receptors (GPCRs) can temporally alter signaling by generating secondary signals from internalized receptors. However, the motifs required to direct GPCRs to these pathways are ill defined. The GPCR, calcium-sensing receptor (CaSR), regulates parathyroid hormone release by promiscuous G protein coupling at cell surfaces and by selectively recruiting Gq/11 to early endosomal membranes. Here, we demonstrate that a dileucine endocytic motif is required for directing CaSR to dynamic spatiotemporal pathways by interacting with the adaptor protein-2 σ-subunit, which is mutated in hypercalcemic patients. From early endosomes, internalized CaSR is targeted to spatially distinct pathways involving early-to-recycling and late endosomes, to direct compartment-specific signals and regulate GPCR forward trafficking. The clinically available allosteric modulator cinacalcet biases signaling to preferentially enhance early endosomal-to-recycling pathways, demonstrating the veracity of targeting discrete endosomal pathways. These results reveal that single GPCRs can simultaneously activate spatially distinct endosomal pathways and demonstrate the structural motifs that facilitate such multidimensional signaling.

## Highlights

• A dileucine endocytic motif directs CaSR to dynamic spatiotemporal signal pathways
• Gαq/11-driven CaSR sustained signaling involves three distinct intracellular sites
• CaSR recruitment to Rab4- and Rab9-positive endosomes enhances sustained signaling
• Cinacalcet acts as a spatially biased allosteric modulator to enhance CaSR signals

## Graphical abstract

**Figure:**
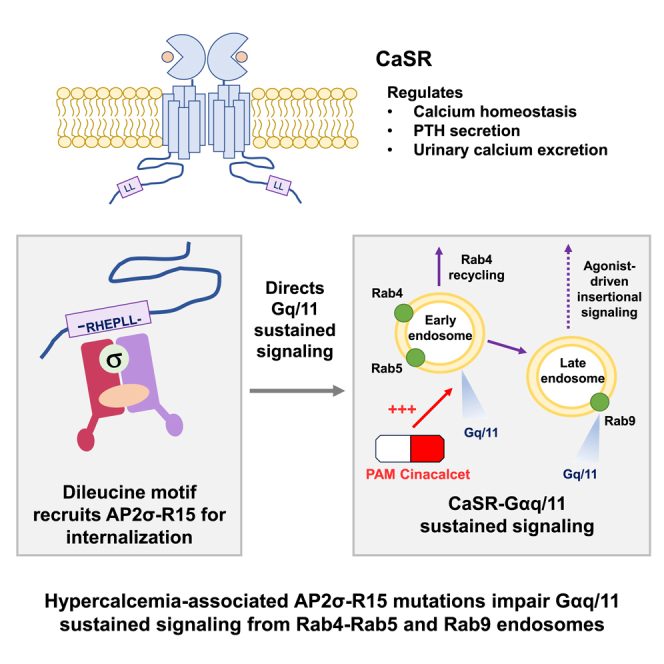

## Introduction

Endosomal signaling has been described for a growing number of G protein-coupled receptors (GPCRs), as a mechanism to alter the temporal profile of G protein signaling, by the generation of a second wave or sustained signal from internalized receptors at endosomes.^1^ Internalization-dependent signaling can drive distinct patterns of gene transcription,^2^ is important for physiological processes, including gut hormone release,^3^ and is a conserved mechanism essential for pheromone signaling in yeast.^4^ Endosomal signaling by GPCRs was initially described for the thyroid-stimulating hormone/thyrotropin receptor and parathyroid hormone receptor (PTH1R) that utilize Gαs and the second messenger cyclic AMP (cAMP).^5^^,^^6^ However, endosomal signaling has since been illustrated for receptors that couple to Gαq, including neurokinin-1 (NK1R), angiotensin II type-1 (AT1R), bradykinin-B2, oxytocin, thromboxane-A2 alpha, and muscarinic acetylcholine-M3 receptors^7^ and Gαi/o, including the free-fatty acid receptor 2 (FFAR2) and opioid receptors (ORs),^3^^,^^8^ and has been extended beyond early endosomes to Golgi^2^ and late endosomes/lysosomes.^9^

For GPCRs that couple to several G proteins, endosomal signaling allows additional complexity, with receptors able to spatially select G proteins with which they couple, allowing complex multidimensional signaling, a phenomenon that may be particularly important for receptors expressed in diverse tissues.^10^ This is illustrated by the calcium-sensing receptor (CaSR), a GPCR that is widely expressed and has roles in extracellular calcium (Ca^2+^_e_) regulation by the parathyroids, kidneys, and bone and non-calcitropic roles including bronchoconstriction, inflammation, and glucose metabolism.^11^^,^^12^^,^^13^ CaSR is activated by multiple endogenous ligands including cations (e.g., Ca^2+^ and Mg^2+^), polyamines (e.g., spermine), and amino acids (e.g., Trp), which bind to orthosteric or allosteric sites in the extracellular domain of the receptor homodimer.^14^ CaSR couples to multiple G protein subtypes of the four subfamilies (Gq/11, Gi/o, G12/13, and Gs)^15^^,^^16^ but predominantly activates Gαi/o, to reduce cAMP signaling, and Gαq/11, stimulating phospholipase C-mediated IP_3_ and mitogen-activated protein kinase pathways (e.g., extracellular signal-related kinase [ERK]1/2).^10^^,^^15^ However, previous studies suggest that internalized CaSR may selectively couple to Gq/11 at intracellular sites to initiate sustained signaling.^10^

CaSR sustained signaling, which occurs in the absence of β-arrestin-1/2, was identified serendipitously while investigating how mutations in the heterotetrameric adaptor protein-2 σ-subunit (AP2σ) that regulates clathrin-mediated endocytosis^17^ impair CaSR signaling in patients with familial hypocalciuric hypercalcemia type-3 (FHH3).^10^ FHH is a disorder of Ca^2+^_e_ homeostasis characterized by lifelong elevations in serum calcium concentrations, normal or increased PTH, and inappropriately low urinary calcium excretion^18^ and is most frequently caused by loss-of-function CaSR mutations (FHH1) but can also be caused by Gα11 (FHH2) and AP2σ mutations.^19^^,^^20^ AP2σ mutations always affect Arg15, which facilitates interactions with dileucine-based endocytic motifs in cargo proteins,^17^ and patients with FHH3 have more marked hypercalcemia and additional features including cognitive dysfunction, compared to patients with FHH1.^18^ *In vitro* studies of these FHH3-associated AP2σ-R15 mutations demonstrated that they decreased CaSR internalization and consequently prevented generation of sustained signals from intracellular membranes.^10^

Despite demonstrating that internalized CaSR can continue signaling from intracellular sites, the structural motifs required for CaSR recognition by AP2σ-R15 and the fate of CaSR following endocytosis are ill defined. Co-immunoprecipitation studies suggested that CaSR binds to AP2σ-R15,^21^ and studies of other membrane proteins would indicate that a CaSR dileucine endocytic motif may govern internalization.^17^ While mutations in a putative C-terminal motif reduces CaSR-mediated Ca^2+^_i_ signaling,^20^ its effect on receptor internalization and sustained signaling is unknown, although such motifs are known to have an important role in membrane protein internalization.^22^ Following internalization, some studies suggest that CaSR is targeted exclusively to degradative pathways including lysosomes and proteasomes^23^^,^^24^^,^^25^; however, others described recycling pathways.^24^^,^^25^ Moreover, few studies used techniques capable of capturing dynamic receptor behavior, a prerequisite when studying such active processes. This is important as CaSR cell surface expression is regulated not only by endocytosis but also an anterograde pathway named agonist-driven insertional signaling (ADIS), in which CaSR is rapidly mobilized to plasma membranes by high [Ca^2+^]_e_.^23^ Additionally, while previous studies indicated CaSR signals at internal sites, the nature of these sites and additional G protein pathways were not investigated.^10^ Thus, a detailed characterization of CaSR trafficking is required to understand the processes governing receptor internalization and the mechanisms by which this patho(physiologically) important receptor mediates sustained endosomal signaling.

Due to its ability to regulate PTH secretion, CaSR has therapeutic potential for the treatment of disorders of calcium homeostasis and osteoporosis. The CaSR-positive allosteric modulator cinacalcet is approved for use in some cases of hyperparathyroidism, although its adverse effects can reduce patient compliance.^26^^,^^27^ Several CaSR-negative allosteric modulators have undergone clinical trials for osteoporosis, although all failed to stimulate new bone formation.^28^ CaSR allosteric modulators affect CaSR trafficking,^29^ although it is not known whether they affect sustained signaling. The ability of drugs to selectively modulate plasma membrane vs. endosomal signaling could provide unique opportunities to achieve tissue-specific targeting and abolish off-target effects. Indeed, several GPCR-targeting drugs, which prolong antinociceptive actions, have already been shown to selectively block sustained signaling of calcitonin receptor-like receptor (CLR) and NK1R.^30^^,^^31^ An understanding of how the current CaSR allosteric modulators affect sustained signaling could inform rational drug design with improved tissue selectivity.

Here, we demonstrate that a dileucine motif in the CaSR cytoplasmic region is required for AP2σ-driven endocytosis and Gαq/11-driven sustained signaling from three intracellular sites. We show that CaSR allosteric modulators have differential effects on sustained signaling, including cinacalcet, which acts as a spatially biased CaSR allosteric modulator.

## Results

### A CaSR C-terminal dileucine internalization motif is important for AP2σ interactions

**Figure 1:**
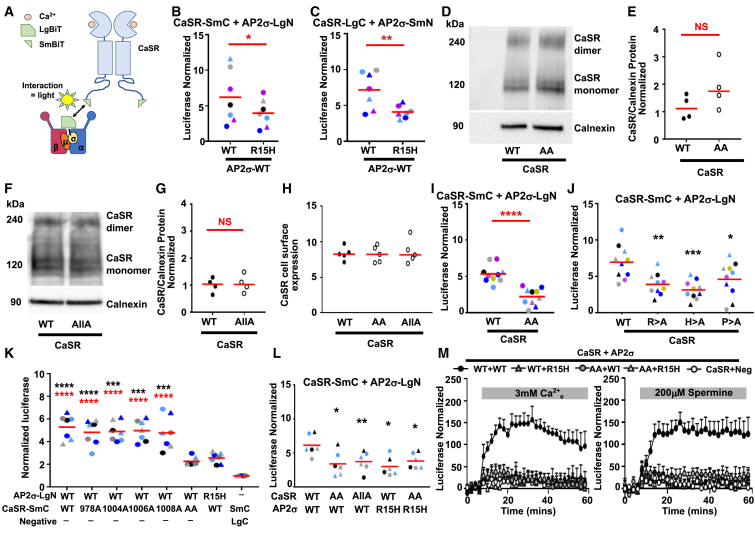
Mutation of the CaSR dileucine motif impairs interactions with AP2σ (A) Schematic depicting the NanoBiT split-luciferase method in which CaSR and AP2σ are tagged with small and large fragments of nanoluciferase (SmBiT and LgBiT). (B and C) NanoBiT luciferase values measured between CaSR and AP2σ-WT or AP2σ-R15H. *N* = 7. (D and E) Western blot of CaSR-WT and CaSR with mutation of residues Leu1013-Leu1014 (CaSR-AA) with (E) densitometry of *N* = 4 blots from independent transfections. (F and G) Western blot of CaSR-WT and CaSR with mutation of residues 1009–1014 with (G) densitometry of *N* = 4 blots from independent transfections. (H) CaSR cell surface expression measured by ELISA in 5 biological replicates. Data are expressed relative to mock-transfected cells. (I) Normalized NanoBiT luciferase values between AP2σ and CaSR-WT or CaSR with alanine mutagenesis of Leu1013-Leu1014 (AA). *N* = 10. (J) Normalized NanoBiT luciferase values measured between AP2σ and mutants of other residues within the CaSR dileucine motif. *N* = 10. (K) Normalized luciferase values between NanoBiT constructs expressing additional alanine mutations within the CaSR C terminus (*N* = 7). Statistics compared to AP2σ-WT+CaSR-AA (black) and to AP2σ-R15H+CaSR-WT (red). (L) Normalized luciferase values between NanoBiT constructs expressing different combinations of AP2σ and CaSR dileucine mutations (*N* = 6). (M) Effect of agonist on NanoBiT luciferase values measured between AP2σ and CaSR dileucine motif mutants. *N* = 5 biological replicates. Values were normalized to cells expressing SmBiT-C-CaSR and the empty LgBiT-C plasmid in NanoBiT assays. Data were analyzed by unpaired t test in (B), (C), (E), (G), and (I), and one-way ANOVA with Sidak’s multiple-comparisons test in (H), (J), and (K). *N* = 5. ∗∗∗∗*p* < 0.0001, ∗∗*p* < 0.01, ∗*p* < 0.05, NS = non-significant compared to WT. Data show mean with each color representing a different replicate. In (M), data shows mean + SEM. Statistical analyses in (J) and (L) compared to WT.

Previous studies proposed a putative dileucine endocytic motif comprising residues 1009 to 1014 (-RHEPLL-) in the CaSR C terminus,^20^ although its effect on CaSR internalization and sustained signaling has not been investigated. We confirmed that this is the only feasible internalization motif using the Eukaryotic Linear Motif prediction tool (Table S1) and subsequently investigated this motif in detail. To determine whether the -RHEPLL- dileucine motif could bind to AP2σ, we used the NanoBiT split-luciferase system with CaSR tagged at the C terminus (Figure 1A). These tags did not affect CaSR signaling or cell surface expression (Figures S1A–S1C), and co-expression of CaSR and AP2σ constructs increased luminescence, indicating that CaSR and AP2σ likely interact (Figures 1B and 1C). Mutation of the LgN-AP2σ-wild-type (WT) or SmN-AP2σ-WT construct to AP2σ-R15H significantly reduced luminescence, consistent with previous co-immunoprecipitation studies^21^ (Figures 1B and 1C). Mutation of either the two leucines at residues 1013–1014 (CaSR-AA) or the whole dileucine motif (CaSR-AllA) did not affect total or cell surface CaSR protein expression in standard media containing ∼2 mM Ca^2+^_e_ (Figures 1D–1H). NanoBiT assays showed that CaSR-AA or three other residues (R1009, H1010, and P1012) of the dileucine motif reduced interactions between CaSR and APσ (Figures 1I and 1J). In contrast, mutations of four other residues not predicted to contribute to the dileucine motif do not affect AP2σ binding (Figure 1K). Mutation of all the residues in the dileucine motif simultaneously reduced luminescence to similar levels as the CaSR-AA (Figure 1L). Addition of either calcium or spermine increased luminescence in cells expressing CaSR-WT and AP2σ-WT, indicating that agonist may enhance interactions but had no effect on luminescence in cells expressing mutant proteins (Figure 1M).

### CaSR dileucine motif mutation impairs internalization

**Figure 2:**
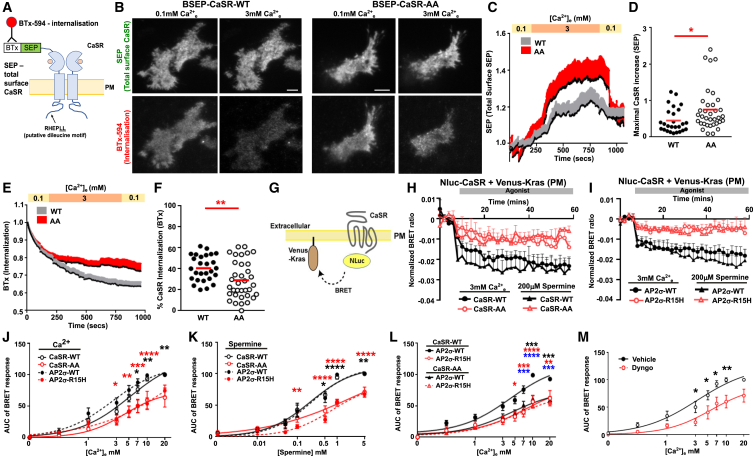
CaSR dileucine motif mutation impairs internalization (A) Schematic of BSEP-CaSR showing the minimal bungarotoxin (BTx)-binding site, which binds BTx-594 to measure endocytosis, and superecliptic pHluorin (SEP), measuring total surface CaSR. (B) Representative TIRF microscopy images of CaSR-WT or CaSR-AA. Scale, 5 μm. (C and D) Quantification of SEP fluorescence with (D) maximal agonist-induced increase. (E and F) Quantification of BTx fluorescence with (F) maximal agonist-induced internalization. *N* = 26 cells (WT), *N* = 35 cells (AA) from *N* = 8 biological replicates. (G) Cartoon showing BRET between CaSR-Nluc and Venus-Kras, a plasma membrane marker. (H and I) Example of BRET between Venus-Kras and (H) CaSR-Nluc-WT or CaSR-Nluc-AA (*N* = 9), and (I) CaSR-Nluc-WT in HEK-AP2σ-WT or HEK-AP2σ-R15H cells (*N* = 6). (J and K) Dose response from BRET responses to (J) Ca^2+^_e_ and (K) spermine between Venus-Kras and CaSR-Nluc or CaSR-AA-Nluc in AP2σ-WT or HEK-AP2σ-R15H. *N* = 5. (L) BRET responses in AP2σ-WT or HEK-AP2σ-R15H cells co-expressing CaSR-Nluc or CaSR-AA-Nluc. *N* = 7. Example data are shown in Figure S3C. (M) BRET examples between Venus-Kras and CaSR-Nluc-WT with vehicle (DMSO) or Dyngo-4a, with concentration response. *N* = 4. Example data are shown in Figure S3D. Data show mean ± SEM, with vehicle subtracted responses for BRET. Statistical analyses were performed using unpaired t test in (D) and (F), two-way ANOVA with Sidak’s multiple comparisons for (J)–(L), one-way ANOVA comparing responses at each concentration in (M). Statistics show CaSR-WT vs. CaSR-AA (black) and AP2σ-WT vs. AP2σ-R15H (red) in (J) and (K). (L) shows AP2σ-WT/CaSR-WT vs. AP2σ-WT/CaSR-AA (black), AP2σ-R15H/CaSR-WT (red), and AP2σ-R15H/CaSR-AA (blue). ∗∗∗∗*p* < 0.0001, ∗∗∗*p* < 0.001, ∗∗*p* < 0.01, ∗*p* < 0.05.

We predicted that the CaSR-AA mutation should impair CaSR trafficking and adopted a similar strategy to that used previously to show that AP2σ-R15 mutations impair endocytosis.^10^ As CaSR cell surface expression is finely balanced between plasma membrane insertion by ADIS and removal by endocytosis, we simultaneously measured these two events using a BSEP-CaSR plasmid.^23^ Endocytosis of cell surface CaSR can be monitored by labeling cells with membrane-impermeable bungarotoxin (BTx)-594 and then measuring cell surface disappearance using total internal reflection fluorescence microscopy (TIRF), while superecliptic pHluorin (SEP) can be used to monitor vesicle fusion (i.e., ADIS) events (Figure 2A). The L1013 and L1014 residues were mutated to alanines to generate BSEP-CaSR-AA and responses compared to cells transfected with BSEP-CaSR-WT. Cells were labeled with BTx-594, then TIRF was immediately performed in imaging buffer with low Ca^2+^_e_ (0.1 mM) for 3 min, followed by 3 mM Ca^2+^_e_ to maximally activate the receptor. In cells transfected with either BSEP-CaSR-WT or BSEP-CaSR-AA, there was an agonist-induced increase in SEP fluorescence, and quantification of the area under the curve (AUC) revealed significantly higher plasma membrane concentrations, with significantly greater maximal responses in CaSR-AA-expressing cells (Figures 2B–2D). Consistent with this elevated plasma membrane CaSR expression, agonist-induced internalization of CaSR was significantly reduced in CaSR-AA-expressing cells (Figures 2B, 2E, and 2F). This indicates that the CaSR dileucine motif is important for agonist-induced internalization.

To provide further evidence that the CaSR dileucine motif and FHH3-associated AP2σ-R15 mutations disrupt internalization, we measured bystander bioluminescence resonance energy transfer (BRET) between CaSR with a C-terminal nanoluciferase tag (CaSR-Nluc) and a Venus-tagged marker of the plasma membrane (Venus-Kras) in live cells (Figure 2G). Addition of Nluc to CaSR plasmids does not significantly affect CaSR expression or signaling (Figures S1C–S1F). We performed BRET assays under several conditions: (1) CaSR-Nluc-WT vs. CaSR-Nluc-AA, (2) CaSR-Nluc-WT in cells stably overexpressing either AP2σ-WT or the FHH3-associated AP2σ-R15H mutant (Figure S2A), which impairs CaSR internalization^10^ but does not affect signaling and trafficking of five other GPCRs (Figures S2B–S2D), and (3) assessment of two different CaSR agonists. We first verified that Kras reliably labeled the plasma membrane and that its expression was not affected by CaSR agonist (Figures S3A and S3B). Exposure of cells to 3 mM Ca^2+^_e_ or 200 μM spermine reduced BRET between CaSR-Nluc and the plasma membrane marker Venus-Kras, consistent with agonist-induced receptor internalization (Figures 2H and 2I). Ligand-induced BRET ratios remained higher in cells expressing CaSR-Nluc-AA and AP2σ-R15H when compared to their respective WT proteins, consistent with impaired internalization (Figures 2H–2K). Expression of CaSR-AA in AP2σ-R15H cells did not have an additive effect on impaired internalization, indicating that AP2σ-R15H acts as a dominant-negative mutant, consistent with previous studies^18^ (Figures 2L and S3C). Higher ligand-induced BRET ratios were also observed in CaSR-Nluc-WT-expressing cells pre-treated with Dyngo-4a, which impairs vesicle scission and reduces endocytosis (Figures 2M, S3D, and S3E).

### Recruitment to early endosomes requires the CaSR dileucine motif and AP2σ-R15

**Figure 3:**
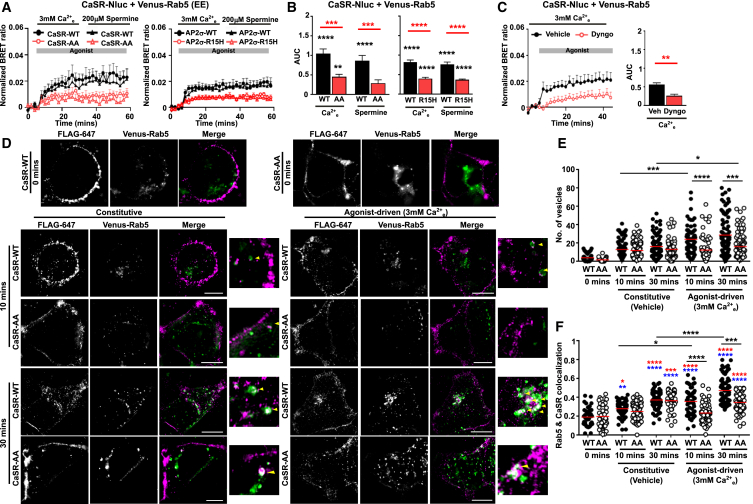
Agonist-induced CaSR trafficking to Rab5 requires the dileucine motif (A and B) BRET between Venus-Rab5 and CaSR-Nluc-WT or CaSR-Nluc-AA (*N* = 9), or CaSR-Nluc-WT in HEK-AP2σ-WT or HEK-AP2σ-R15H cells (*N* = 7), with (B) AUC. (C) BRET between Venus-Rab5 and CaSR-Nluc-WT with vehicle (DMSO) or Dyngo-4a. *N* = 5. (D) Example SIM images of FLAG-CaSR-WT or FLAG-CaSR-AA with Venus-Rab5. Close-up images are shown to the right. Cells were exposed to FLAG antibody and vehicle or 3 mM Ca^2+^_e_ for 0, 10, or 30 min. Scale, 5 μm. Arrows show colocalization. Data shows mean + SEM in A–C. (E and F) Quantification of the total number of vesicles and (F) colocalization measured by Pearson’s coefficient between FLAG-CaSR-WT or FLAG-CaSR-AA and Venus-Rab5. Number of cells from four biological replicates were as follows: WT vehicle 0 min (47), WT vehicle 10 min (76), WT vehicle 30 min (86), WT 3 mM Ca^2+^_e_ 10 min (66), WT 3 mM Ca^2+^_e_ 30 min (89), AA vehicle 0 min (50), AA vehicle 10 min (88), AA vehicle 30 min (75), AA 3 mM Ca^2+^_e_ 10 min (77), AA 3 mM Ca^2+^_e_ 30 min (99). Comparisons of each to WT or AA at 0 min were *p* < 0.0001 in (D). Comparisons to WT or AA at 0 min are shown in blue and red, respectively. Red bar shows median in E and F. Statistical analyses were performed using one-way ANOVA with Tukey’s multiple-comparisons test for (B), unpaired t test in (C), and Kruskal-Wallis one-way ANOVA with Dunn’s multiple-comparisons test in (E) and (F). ∗∗∗∗*p* < 0.0001, ∗∗∗*p* < 0.001, ∗*p* < 0.05.

To determine where CaSR traffics to following endocytosis, we measured agonist-induced bystander BRET between CaSR and Venus-tagged markers of several subcellular compartments. There were robust agonist-induced BRET responses between CaSR-Nluc and Venus-Rab5, a marker of early endosomes, consistent with previous studies that showed CaSR colocalizes with Rab5 following receptor activation^10^ (Figures 2A and 2B). These ligand-induced responses were impaired by CaSR-Nluc-AA, AP2σ-R15H, and Dyngo-4a when compared to their respective WT proteins, consistent with impaired internalization (Figures 2A–2C), and highly inclined and laminated optical sheet (HILO) imaging showed agonist-induced Rab5 co-localization with CaSR (Figure S4A). To investigate how important the dileucine motif is to internalization to Rab5 pathways, we performed structured illumination microscopy (SIM) imaging of FLAG-CaSR-WT or FLAG-CaSR-AA and assessed receptor recruitment to Rab5-containing endosomes in the presence of vehicle (to assess constitutive endocytosis) and agonist (to assess agonist-driven internalization). This showed similar constitutive internalization in the two cell types (total number of vesicles and CaSR-Rab5 colocalization were not significantly different between the two groups) (Figures 3D–3F), indicating that the dileucine motif may not be required for constitutive internalization. In contrast, cells expressing FLAG-CaSR-AA had significantly fewer CaSR-positive internalized vesicles, and colocalization between CaSR-AA and Rab5 was significantly reduced when compared to FLAG-CaSR-WT-expressing cells (Figures 3D–3F). Therefore, the dileucine motif is important for CaSR agonist-driven internalization.

**Figure 4:**
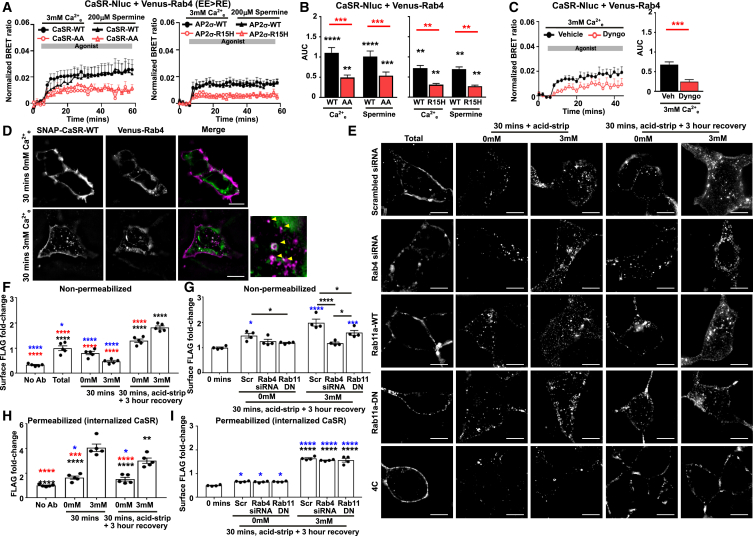
CaSR traffics to Rab4 pathways and recycles by Rab4 and Rab11 (A and B) BRET between Venus-Rab4 and CaSR-Nluc-WT or CaSR-Nluc-AA (*N* = 7), or CaSR-Nluc-WT in HEK-AP2σ-WT or HEK-AP2σ-R15H cells (*N* = 9), with (B) AUC. (C) BRET between Rab4-Venus and CaSR-Nluc-WT, with vehicle (DMSO) or Dyngo-4a. *N* = 5. (D) SIM of SNAP-CaSR-647 and Venus-Rab4. Scale, 5 μm. Arrows show colocalization. *N* = 23 (0 mM), *N* = 22 (3 mM) from *N* = 4 biological replicates. (E) SIM images of cells exposed to FLAG antibody and either non-permeabilized (total) or exposed to 0 or 3 mM Ca^2+^_e_ for 30 min or 30 min with 3-h recovery, before acid strip to remove cell surface FLAG. Internalization occurs in cells stimulated with 3 mM Ca^2+^_e_ conditions other than plates incubated at 4°C to block endocytosis. Cells transfected with Rab4 siRNA and Rab11-DN have reduced receptor recycling (seen as FLAG expression at cell surfaces) than cells transfected with scrambled siRNA or Rab11-WT. Scale, 5 μm. (F) Non-permeabilized cells incubated with FLAG antibody and exposed to 0 or 3 mM Ca^2+^_e_ for 30 min or 30 min with 3-h recovery to monitor recycling. Comparisons to 3 mM (30 min) in black, to 3 mM (30 min with recovery) in red, and to 0 mM (30 min with recovery) in blue. *N* = 5. (G) Antibody feeding in cells transfected with scrambled siRNA, Rab4 siRNA, or Rab11a-dominant negative (DN). Comparisons to 0 min (blue). *N* = 5. (H) Antibody feeding assays in which permeabilized cells were incubated with FLAG antibody and exposed to 0 or 3 mM Ca^2+^_e_ for 30 min or 30 min with 3-h recovery. Statistics show comparisons to 3 mM (30 min) in black, to 3 mM (30 min with recovery) in red, and to 0 mM (30 min with recovery) in blue. *N* = 5. (I) Antibody feeding assays in cells transfected with scrambled siRNA, Rab4 siRNA, or Rab11a-DN and permeabilized to monitor total CaSR. Statistical analyses show comparisons to 0 min in blue and between 0 and 3 mM in each condition in black. *N* = 4. Statistical analyses were performed using one-way ANOVA with Tukey’s multiple-comparisons test for (B), unpaired t test in (C), and one-way ANOVA with Sidak’s multiple-comparisons test in (F)–(I). ∗∗∗∗*p* < 0.0001, ∗∗∗*p* < 0.001, ∗∗*p* < 0.01, ∗*p* < 0.05. Data shows mean + SEM in A–C,and F–I.

Ca^2+^_e_ increased BRET responses between CaSR and Rab4, which also localizes to early endosomes (Figures 4A and 4B). Recruitment to Rab4-positive endosomes was similarly impaired by CaSR-Nluc-AA, AP2σ-R15H, and Dyngo-4a (Figures 4A–4C). To verify CaSR trafficking to Rab4 vesicles, SIM imaging was performed in cells exposed to 0 or 5 mM Ca^2+^_e_ for 30 min. Colocalization increased in cells exposed to 3 mM Ca^2+^_e_ (Pearson’s coefficient: 0.21 ± 0.02 [0 mM] and 0.30 ± 0.02 [5 mM] for 30 min, *p* < 0.001) (Figure 4D). HILO imaging, which is lower resolution but can be performed in live cells over prolonged periods, similarly showed an increase in colocalization between CaSR and Rab4-containing vesicles (Figure S4B), while no colocalization was observed between an unrelated GPCR (the ghrelin receptor) and Rab4 upon addition of 3 mM Ca^2+^_e_ to cells (Figure S4C), indicating that CaSR and Rab4 colocalization is likely driven by CaSR activation.

Rab4 has a role in trafficking receptors to recycling pathways, and we next determined whether Rab4 contributes to CaSR trafficking to plasma membranes. We performed antibody feeding experiments to monitor FLAG-labeled CaSR upon stimulation with 0 or 3 mM Ca^2+^_e_, or stimulation followed by a 3-h recovery in basal HBSS to observe recycling by SIM (Figure 4E) and quantified expression in plate assays (Figures 4F–4I). We assessed the effect of disruption of Rab4 after first showing that Rab4 small interfering RNA (siRNA) successfully reduces Rab4 protein, has no overall effect on vesicular trafficking of transferrin, and CaSR still internalizes (Figures S5A–S5C), indicating it is unlikely to have off-target effects. We also assessed the effect of overexpression of a dominant-negative form of Rab11a as a previous study showed that CaSR may utilize Rab11 for recycling.^24^ CaSR stimulation increased receptor expression at cell surfaces following cell recovery (i.e., increased receptor recycling) (Figures 4E–4G). Recycling was impaired by disruption of Rab4 and Rab11, although total CaSR in permeabilized cells was similar (Figures 4E–4I). Therefore, CaSR undergoes some receptor recycling by both Rab4 and Rab11 pathways.

### CaSR traffics to Rab9 vesicles and facilitates release from the Golgi pathway

**Figure 5:**
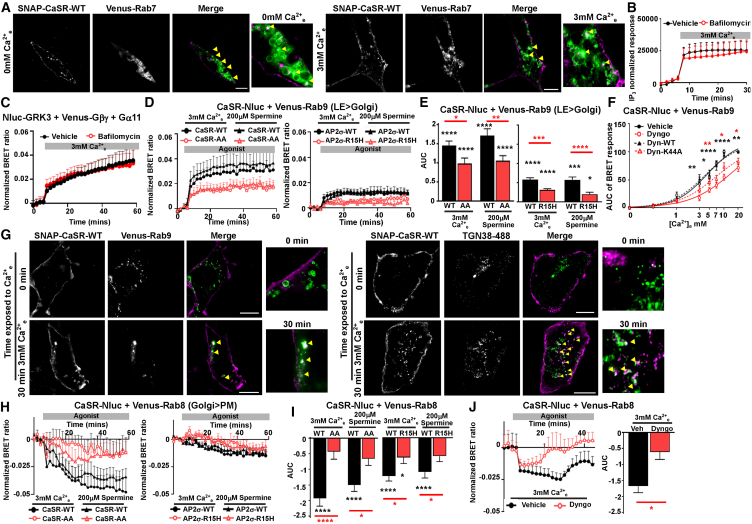
CaSR activation stimulates trafficking to Rab9 vesicles and facilitates receptor release from the Golgi (A) SIM of SNAP-CaSR-647 and Venus-Rab7 exposed to 0 or 3 mM Ca^2+^_e_. Scale, 5 μm. *N* = 50 (0 mM), *N* = 60 (3 mM) from *N* = 4 biological replicates. (B and C) NanoBiT IP3 biosensor responses (*N* = 4) and (C) BRET between Nluc-GRK3 and Venus-Gβγ in cells overexpressing Gα11 (*N* = 4), with vehicle or bafilomycin. (D and E) BRET between Venus-Rab9 and CaSR-Nluc-WT or CaSR-Nluc-AA, or CaSR-Nluc-WT in HEK-AP2σ-WT or HEK-AP2σ-R15H cells, with (E) AUC. *N* = 8. Black asterisk compared to vehicle. (F) BRET between CaSR-Nluc and Venus-Rab9 with vehicle (DMSO) or Dyngo, or in cells overexpressing dynamin-WT or dynamin-K44A. *N* = 5. Statistics: vehicle vs. Dyngo-4a (black), Dynamin-WT vs. Dynamin-K44A (red). (G) SIM of SNAP-CaSR-WT and Venus-Rab9 or TGN38-488 following exposure to agonist for 0 or 30 min. *N* = 26 (0 mM), *N* = 21 (5 mM) from 3 biological replicates (Rab9). *N* = 14 (0 mM), *N* = 12 (5 mM) from 3 biological replicates (TGN38). Arrows show colocalization. (H and I) BRET between Venus-Rab8 and CaSR-Nluc-WT or CaSR-Nluc-AA (*N* = 8), or CaSR-WT-Nluc in HEK-AP2σ-WT or HEK-AP2σ-R15H cells (*N* = 7), with (I) AUC. (J) BRET between Venus-Rab8 and CaSR-Nluc-WT with vehicle or Dyngo-4a, with AUC. *N* = 4. BRET shows vehicle subtracted responses with mean ± SEM. Scale, 5 μm in all images. Statistical analyses were performed by one-way ANOVA with Tukey’s multiple-comparisons test for (E) and (I), two-way ANOVA comparing responses at each agonist concentration with Sidak’s multiple-comparisons test in (F), and unpaired t test in (J). ∗∗∗∗*p* < 0.0001, ∗∗∗*p* < 0.001, ∗∗*p* < 0.01, ∗*p* < 0.05.

Previous studies indicated that CaSR is trafficked to degradation pathways that may involve lysosomes or proteasomes.^23^ To determine whether agonist-driven CaSR is trafficked to the lysosome, we performed SIM in cells expressing SNAP-CaSR and Venus-Rab7, a late endosome-to-lysosome marker. This confirmed some colocalization, although there was no significant difference between cells exposed to 0 or 3 mM Ca^2+^_e_ for 30 min (Pearson’s coefficient: 0.33 ± 0.03 [0 mM], 0.38 ± 0.02 [5 mM], *p* = 0.19) (Figure 5A). Moreover, CaSR functional responses, measured by independent assays assessing G protein activity and downstream signaling, were not affected by exposure of cells to bafilomycin-A1, which reduces lysosomal acidification (Figures 5B and 5C). In contrast, bafilomycin treatment of cells transfected with other GPCRs known to be targeted to lysosomal pathways^32^^,^^33^ did have a significant effect on signaling (Figures S6A and S6B). Therefore, trafficking to lysosomes is unlikely to be a major trafficking pathway for CaSR, and internalized receptor may be targeted to other degradation pathways (e.g., proteosomes).

To determine whether CaSR is trafficked to other intracellular organelles from the endocytic pathway, we examined BRET interactions between CaSR and Rab9, a marker of late endosome-to-Golgi pathways.^34^ This demonstrated robust agonist-induced increases in BRET between CaSR and Rab9 (Figures 5D and 5E). This was impaired by CaSR dileucine motif or AP2σ-R15 mutations and by pre-incubation with Dyngo-4a or upon overexpression of dynamin-K44A, indicating that CaSR trafficking to a Rab9-positive pathway is dependent on internalization (Figure 5F). Pre-incubation with brefeldin A or cycloheximide did not significantly affect CaSR trafficking to Rab9, providing further evidence that the receptor is from the endocytic pathway or from pre-formed vesicles, rather than newly synthesized from the endoplasmic reticulum (ER) (Figures S6C and S6D). SIM and HILO confirmed an agonist-driven increase in colocalization between CaSR and Rab9 (Pearson’s coefficient: 0.28 ± 0.03 [0 mM], 0.37 ± 0.02 [3 mM], *p* = 0.0091), as well as the *trans*-Golgi marker TGN38 (Pearson’s coefficient: 0.20 ± 0.04 [0 mM], 0.35 ± 0.03 [3 mM], *p* = 0.0075) (Figures 5G and S6E).

To further investigate whether CaSR is trafficked to a pathway involving the Golgi, we examined two additional markers of the ER-Golgi pathway, Rab8 as a marker of Golgi-to-plasma membrane exocytic events and Rab1 as a marker of ER-to-Golgi. There was a significant reduction in BRET between CaSR and Rab8 upon ligand stimulation, consistent with forward trafficking to the plasma membrane (i.e., ADIS) (Figures 5H and 5I). The CaSR dileucine mutant, AP2σ-R15 mutant, and Dyngo-4a reduced BRET between CaSR and Rab8 (Figures 5H–5J), indicating that CaSR endocytosis, or signals following internalization, contributes to ADIS. In contrast, there was no significant change in BRET between Rab1 and CaSR (Figures S7A and S7B). No trafficking to mitochondria was observed (Figures S7C–S7F).

### Mutation of the CaSR dileucine motif impairs sustained signaling

**Figure 6:**
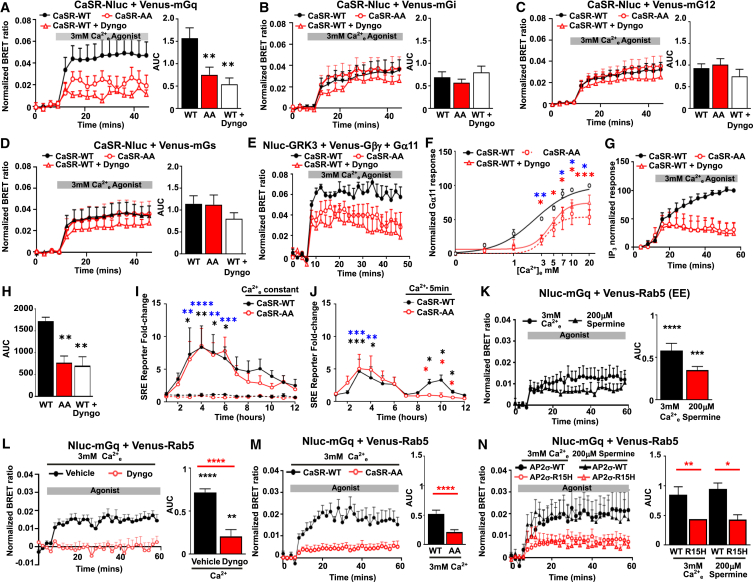
Mutation of CaSR dileucine motif impairs Gα_q/11_ signaling (A–D) BRET between CaSR-Nluc and (A) Venus-mGq, (B) Venus-mGi, (C) Venus-mG12, (D) Venus-mGs. *N* = 7. Asterisks compared to WT. (E) Example BRET response between Nluc-GRK3 and Venus-Gβγ in cells expressing Gα11 and CaSR-WT or CaSR-AA, with vehicle or Dyngo-4a. (F) Dose response of BRET responses. Statistics: WT vs. AA (red), WT vs. WT with Dyngo-4a (blue). *N* = 5. (G and H) NanoBiT IP_3_ responses in CaSR-WT or CaSR-AA with vehicle or Dyngo-4a, with (H) AUC. *N* = 4. (I) SRE luciferase reporter responses to 0.1 mM (dotted lines) or 3 mM (solid lines) Ca^2+^_e_ over 12 h in cells expressing CaSR-WT or CaSR-AA. *N* = 4. (J) SRE luciferase responses to a 5-min pulse of 3 mM Ca^2+^_e_. *N* = 4. Asterisks show WT 0.1 mM vs. 5 mM Ca^2+^_e_ (black), AA 0.1 mM vs. 5 mM Ca^2+^_e_ (blue), and WT vs. AA (red). (K–N) BRET between Venus-Rab5 and Nluc-mGq with: (K) calcium (*N* = 8) or spermine (*N* = 5), (L) with vehicle (DMSO) or Dyngo-4a (*N* = 4), (M) with CaSR-WT or CaSR-AA (*N* = 4), or (N) in HEK-AP2σ-WT or HEK-AP2σ-R15H cells (*N* = 4). Statistical analyses were performed using two-way ANOVA with Sidak’s test in (F), (I), and (J) comparing responses between groups at each concentration, one-way ANOVA with Sidak’s test in (A)–(D), and with Tukey’s test in (H), (K), (M), and (N), and unpaired t test for (L). ∗∗∗∗*p* < 0.0001, ∗∗∗*p* < 0.001, ∗∗*p* < 0.01, ∗*p* < 0.05. Data shows mean + SEM.

Our previous studies showed that CaSR can continue signaling once internalized and that this sustained signaling is impaired by FHH3-associated AP2σ-R15 mutations.^10^ As the CaSR-AA mutation has a similar effect on endocytosis, we hypothesized that this mutation would similarly impair sustained signaling. To investigate how the CaSR dileucine mutation may affect CaSR signaling, we first assessed the recruitment of mini-G proteins to CaSR-Nluc-WT and CaSR-Nluc-AA using BRET.^35^ Recruitment of mini-Gq (a Gs/q chimera containing residues of the α5 helix shared in Gα11 and Gαq^36^) to Nluc-CaSR-AA was significantly impaired when compared to CaSR-Nluc-WT (Figure 6A). Pre-treatment of cells expressing CaSR-Nluc-WT with Dyngo-4a similarly impaired mGq recruitment (Figure 6A). In contrast, the CaSR dileucine motif mutant and pre-treatment with Dyngo-4a did not affect recruitment of mini-Gi, -G12, or Gs (Figures 6B–6D). Thus, CaSR-AA and Dyngo-4a preferentially suppress CaSR-mediated recruitment of Gq.

It is possible that mutation of the CaSR dileucine motif may impair Gαq recruitment but have no effect on downstream signaling, or may not affect recruitment of other G proteins but could still impact their downstream signaling. We therefore assessed signaling using a variety of assays in cells expressing pcDNA-FLAG-CaSR-WT or pcDNA-FLAG-CaSR-AA plasmids. Firstly, we used the Nluc-GRK3 and Venus-Gβγ assay to measure Gα11 signaling and showed impaired responses in CaSR-AA-expressing cells (Figures 6E and 6F). BRET responses were similarly impaired in CaSR-WT cells pre-treated with Dyngo-4a (Figure 6F). CaSR-AA also impaired IP_3_ responses to similar levels as Dyngo-4a-treated CaSR-WT cells in NanoBiT IP3 biosensor assays (Figures 6G and 6H). In contrast, Dyngo-4a did not significantly reduce CaSR-AA responses in any assays (Figures S8A–S8C). Activation of Gαq/11 also stimulates phosphorylation of ERK1/2 (p-ERK1/2), and we have previously shown that CaSR stimulates a rapid increase in p-ERK1/2 within 2–5 min, while a second wave of sustained signaling occurs at ∼30 min post stimulation and is sensitive to Dyngo-4a and the AP2σ-R15 mutation.^10^ Using western blot analysis, CaSR-AA was similarly shown to stimulate rapid signaling (2 min after stimulation), but the sustained signal at 30–60 min was not observed (Figure S8D). Moreover, sustained SRE luciferase reporter responses (9–10 h) were similarly impaired, while immediate responses (within 3–4 h) were unaffected (Figures 6I and 6J). CaSR-AA did not affect Fsk-induced cAMP responses, providing further evidence that CaSR internalization has no effect on Gi/o-cAMP signaling (Figures S8E and S8F).

### CaSR sustained signaling is mediated by Gαq/11

To identify which membranes are involved in CaSR sustained signaling and verify which G protein pathways can elicit these signals, we next investigated the CaSR-mediated recruitment of Nluc-tagged mini-G proteins to Venus-tagged membranes using bystander BRET. There was no recruitment of mini-G proteins to Venus-Kras, a cell surface marker, in the absence of receptor (Figure S9). When CaSR was expressed, there was robust recruitment of all four mini-G proteins to Kras-labeled plasma membranes with two CaSR agonists (Figures S10A–S10C). CaSR activation mediated the recruitment of mini-Gq to Rab5-labeled endosomes, shown by BRET and HILO (Figures 6K, S10D, and S10E). Recruitment of the full-length GFP-labeled Gα11 was also investigated by HILO (Figures S10F and S10G). This showed a significant increase in colocalization between Gα11 and Rab5 following exposure to agonist (Pearson’s coefficient: 0.57 ± 0.01 [0 min], 0.61 ± 0.01 at 10 min, *p* < 0.02; 0.63 ± 0.02 at 15 min, *p* < 0.01). Recruitment of mini-Gq or Gα11 to Rab5-positive endosomes was not observed in cells exposed to vehicle. Ligand-stimulated recruitment of mini-Gq was impaired by Dyngo-4a (Figures 6L and S10H). Moreover, Gαq recruitment to Rab5-positive early endosomes was reduced in cells overexpressing the CaSR dileucine motif and AP2σ-R15 mutants (Figures 6M and 6N). CaSR activation with calcium or spermine did not recruit mini-Gi, -G12, or -Gs to Rab5-containing endosomes when measured by BRET (Figures S11A–S11C) or imaging (Figures S11D and S11E), and Dyngo-4a did not affect these responses (Figures S11F–S11H). Control experiments showed that BRET could detect the recruitment of Gi and Gs to Rab5 endosomes when other GPCRs were activated (Figures S11I and S11J). Additionally, pre-incubation of cells with Dyngo-4a had no effect on either Gi-mediated suppression of forskolin-induced cAMP or on agonist-induced increases in cAMP (Figures S11K and S11L). Thus, CaSR sustained signaling is mediated by the Gαq/11 signaling pathway and requires the CaSR dileucine motif and AP2σ-R15.

### CaSR signaling from Rab4- and Rab9-positive endosomes contributes to sustained signaling

**Figure 7:**
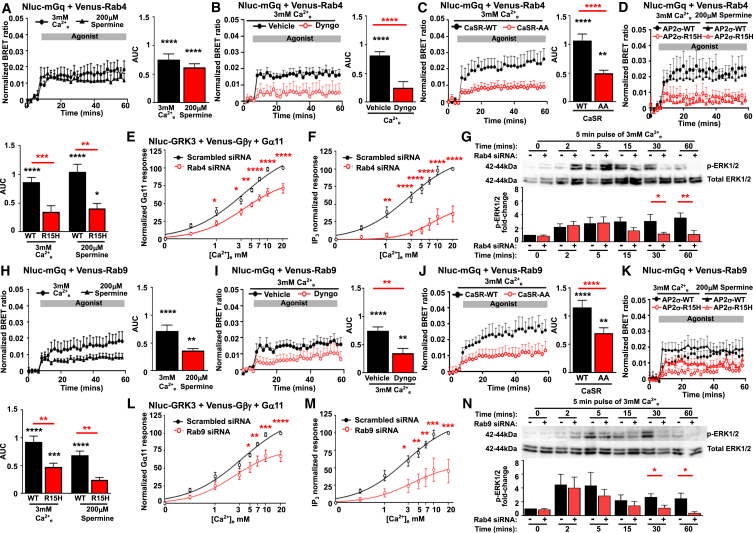
CaSR signaling from Rab4- and Rab9-positive endosomes contributes to sustained signaling (A) BRET between Venus-Rab4 and Nluc-mGq in HEK-CaSR. Calcium (*N* = 9), spermine (*N* = 6). (B) Ca^2+^-induced BRET with vehicle (DMSO) or Dyngo-4a. *N* = 8. (C and D) BRET between Venus-Rab4 and CaSR-Nluc-WT or CaSR-Nluc-AA or (D) CaSR-Nluc-WT in HEK-AP2σ-WT or HEK-AP2σ-R15H cells. *N* = 7. (E) AUC of ligand-induced BRET between Nluc-GRK3 and Venus-Gβγ in HEK-CaSR overexpressing Gα11, with scrambled or Rab4 siRNA. *N* = 8. (F) AUC of NanoBiT IP_3_ responses with scrambled or Rab4 siRNA. *N* = 6. (G) Western blot of *p*-ERK1/2 in HEK-CaSR with scrambled or Rab4 siRNA following a 5-min pulse of 3 mM Ca^2+^ then 0.1 mM Ca^2+^. Quantification of densitometry from five western blots is shown below. (H) BRET between Venus-Rab9 and Nluc-mGq in HEK-CaSR. Calcium (*N* = 9), spermine (*N* = 6). (I) BRET with vehicle (DMSO) or Dyngo-4a. *N* = 8. (J and K) Ca^2+^-induced BRET in (J) cells transfected with CaSR-Nluc-WT or CaSR-Nluc-AA (*N* = 5) or in (K) HEK-AP2σ-WT or HEK-AP2σ-R15H cells (*N* = 10). (L) BRET between Nluc-GRK3 and Venus-Gβγ in HEK-CaSR overexpressing Gα11, with scrambled or Rab9 siRNA. *N* = 8. (M) NanoBiT IP_3_ responses with scrambled or Rab9 siRNA. *N* = 6. (N) *p*-ERK1/2 responses with scrambled or Rab4 siRNA and exposed to a 5-min pulse of 3 mM Ca^2+^_e_. Quantification of densitometry from four western blots is shown below. Data shows mean ± SEM. ∗∗∗∗*p* < 0.0001, ∗∗∗*p* < 0.001, ∗∗*p* < 0.01, ∗*p* < 0.05. Black asterisks show vehicle vs. agonist. Statistical analyses were performed using one-way ANOVA with Sidak’s multiple comparisons test for (A), (B), (C), (D), (G), (H), (I), (J), (K), and (N), and two-way ANOVA with Sidak’s test for (E), (F), (L), and (M).

We next determined whether Gq is recruited to the other membranes that CaSR is trafficked to following internalization. A robust BRET signal was observed between Gαq and Rab4 upon stimulation with two CaSR agonists (Figure 7A). This recruitment was sensitive to Dyngo-4a and impaired in cells expressing CaSR-AA and AP2σ-R15H (Figures 7B–7D). Rab4 siRNA significantly reduced Gα11 activation (measured by GRK3-split-Gβγ, Figures 7E and S12A) and impaired IP_3_ responses, when compared to scrambled siRNA (Figures 7F and S12B). Rab4 siRNA also reduced p-ERK1/2 sustained responses measured by immunoblot and AlphaScreen assays (Figures 7G and S12C). Thus, a Rab4 pathway may be involved in sustained signaling, although as Rab4 siRNA reduces mGq recruitment and CaSR recruitment (Figures S12D–S12G) to Rab5-containing endosomes, it is possible that reduced signaling observed in assays with Rab4 siRNA could be due in part to reduced recruitment to Rab5-positive endosomes.

Studies were then performed in the presence of Rab9 siRNA, which we showed successfully reduced Venus-Rab9 protein (Figure S5A). Trafficking of transferrin and internalization of CaSR were unaffected by Rab9 siRNA (Figures S5B and S5C). CaSR stimulation increased BRET between mini-Gq and Rab9, which was reduced by Dyngo-4a, CaSR-AA, and AP2σ-R15H (Figures 7H–7K). CaSR signaling was also reduced in cells transfected with Rab9 siRNA (Figures 7L, 7M, S12I, and S12J). Moreover, Rab9 siRNA reduced p-ERK1/2 sustained responses similarly to Rab4 siRNA and CaSR-AA (Figures 7N and S12C). No significant changes in BRET were observed between mini-Gq and other Venus-tagged markers, indicating that sustained signaling is unlikely to involve these pathways (Figure S13). Moreover, recruitment of mGi and mG12 to other Venus-tagged markers was not observed in HEK-CaSR cells providing further evidence that sustained signaling is preferentially mediated by the Gαq/11 pathway (Figure S13).

### Allosteric modulators enhance CaSR sustained signaling

**Figure 8:**
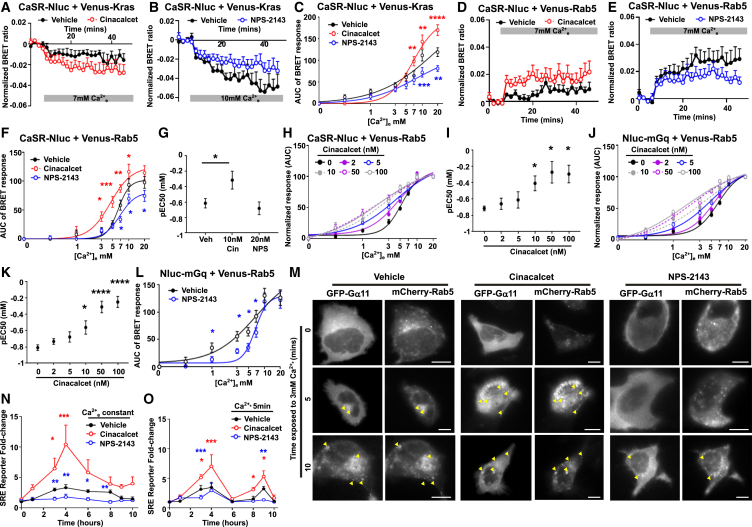
Sustained signaling can be regulated by CaSR allosteric modulators (A–C) BRET between Venus-Kras and CaSR-Nluc with vehicle or (A) 10 nM cinacalcet, (B) 20 nM NPS-2143, with (C) dose response of BRET responses. *N* = 6. (D and E) BRET between Venus-Rab5 and CaSR-Nluc with vehicle or (D) 10 nM cinacalcet (*N* = 5), (E) 20 nM NPS-2143 (*N* = 6). (F and G) Dose response of BRET responses, with (G) pEC50 values. (H and I) BRET between Nluc-mGq and Venus-Rab5 with increasing cinacalcet concentrations, with (I) pEC50. *N* = 4. (J and K) BRET between Nluc-mGq and Venus-Rab5 with increasing concentrations of cinacalcet with (K) pEC50. (L) BRET between Nluc-mGq and Venus-Rab5 with 20 nM NPS-2143. *N* = 12. (M) HILO images from HEK-CaSR cells transfected with GFP-Gα11 and mCherry-Rab5. *N* = 20 cells (vehicle), *N* = 17 cells (cinacalcet) from 4 biological replicates, *N* = 26 cells (NPS-2143) from 5 biological replicates. Scale bar, 5 μm. Arrows show colocalized spots. (N) SRE luciferase reporter activity to 3 mM Ca^2+^_e_ in HEK-CaSR cells pre-treated with vehicle, 10 nM cinacalcet, and 20 nM NPS-2143. *N* = 4. (O) SRE luciferase reporter responses to a 5-min pulse of 3 mM Ca^2+^_e_. *N* = 4. Statistics show vehicle vs. cinacalcet (red), vehicle vs. NPS-2143 (blue). Data show mean ± SEM. Statistical analyses used two-way ANOVA with Dunnett’s test in (C) and (F), Tukey’s test for (L), and Sidak’s test in (N) and (O). (G), (I), and (K) used one-way ANOVA with Dunnett’s test and show comparisons to 0 nM cinacalcet. Data in (A), (B), (D), and (E) show representative BRET data used to generate dose-response curves. ∗∗∗∗*p* < 0.0001, ∗∗∗*p* < 0.001, ∗∗*p* < 0.01, ∗*p* < 0.05.

Finally, the effect of CaSR-positive and negative allosteric modulators (PAMs, NAMs) on CaSR sustained signaling was investigated. Trafficking of Nluc-CaSR to Venus-tagged intracellular membranes was assessed with the PAM cinacalcet (10 nM) and the NAM NPS-2143 (20 nM). Cinacalcet increased CaSR trafficking at high concentrations of agonist, while the NAM impaired trafficking (Figures 8A–8C). Cinacalcet increased BRET, while NPS-2143 reduced BRET between CaSR and Rab5, consistent with enhanced and impaired trafficking to early endosomes, respectively (Figures 8D–8G). Cinacalcet enhanced responses in a dose-dependent manner with pEC50 values increased until saturation at 50 nM cinacalcet (Figures 8H and 8I), consistent with PAM effects. In the presence of cinacalcet, agonist-mediated recruitment of mGq to Rab5-positive early endosomes was increased, while NPS-2143 reduced recruitment (Figures 8J and 8L). HILO imaging showed that the PAM rapidly recruited Gα11 to Rab5-positive endosomes, with a greater colocalization observed between the two markers when compared to vehicle-treated cells at multiple time points (Figure 8M, Pearson’s coefficient: vehicle 0.59 ± 0.01 vs. cinacalcet 0.63 ± 0.03, *p* < 0.03 at basal; 0.61 ± 0.01 vs. 0.64 ± 0.006 at 5 min, *p* < 0.04; 0.62 ± 0.01 vs. 0.65 ± 0.007 at 10 min, *p* < 0.02). Conversely, the NAM reduced Gα11 recruitment to Rab5-positive endosomes (Figure 8M, Pearson’s coefficient: NPS-2143 0.57 ± 0.01 at basal, not significant; 0.57 ± 0.01 at 5 min, *p* < 0.047; 0.058 ± 0.01 at 10 min, *p* < 0.05; *p* < 0.37). Cinacalcet increased agonist-driven SRE luciferase activity, while NPS-2143 reduced signaling in the presence of constant calcium (Figure 8N). Furthermore, cinacalcet increased sustained SRE signals, while NPS-2143 abolished these effects, consistent with their effects on recruitment of Gα11 to early endosomes (Figure 8O).

**Figure 9:**
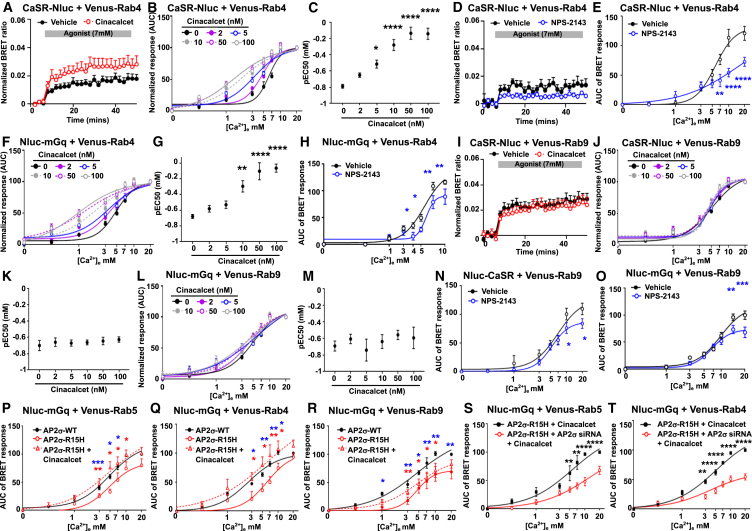
Cinacalcet increases CaSR signaling in FHH3-associated AP2σ-R15H mutant cells by enhancing sustained signaling (A) BRET between Venus-Rab4 and CaSR-Nluc with vehicle or cinacalcet. (B and C) Dose response of BRET responses with increasing cinacalcet concentrations with (C) pEC50 values. *N* = 4. (D and E) BRET between Venus-Rab4 and CaSR-Nluc with vehicle or NPS-2143, with (E) dose response. *N* = 6. (F and G) BRET between Venus-Rab4 and Nluc-mGq with increasing cinacalcet concentrations, with (G) pEC50. *N* = 4. (H) BRET between Nluc-mGq and Venus-Rab4 with NPS-2143. *N* = 12. (I–K) BRET between Nluc-mGq and Venus-Rab9 with cinacalcet, with (J) increasing cinacalcet concentrations, and (K) pEC50. *N* = 5. (L and M) BRET between Nluc-mGq and Venus-Rab9 in cells with increasing concentrations of cinacalcet, with (M) pEC50. *N* = 5. (N) BRET between Nluc-CaSR and Venus-Rab9 with NPS-2143. *N* = 12. (O) BRET between Nluc-mGq and Venus-Rab9 with NPS-2143. *N* = 11. (P–R) BRET between Nluc-mGq and (P) Rab5 (*N* = 6), (Q) Rab4 (*N* = 4), (R) Rab9 (*N* = 5), in AP2σ-WT or AP2σ-R15H cells expressing CaSR with vehicle or cinacalcet. (S and T) Cinacalcet effect on responses in cells expressing a siRNA-resistant AP2σ-R15H plasmid combined with AP2σ-siRNA to knockdown endogenous protein or an AP2σ-R15H plasmid. *N* = 5. Statistics show: WT vs. AP2σ-R15H (red), AP2σ-R15H with vehicle or cinacalcet (blue) in (P)–(R). Statistical analyses were performed using two-way ANOVA with Dunnett’s test in (E), and Sidak’s test in (H,) (N), (O), (P), (Q), (R), (S), and (T), and one-way ANOVA with Dunnett’s test in (C), (G), (K), and (M). ∗∗∗∗*p* < 0.0001, ∗∗∗*p* < 0.001, ∗∗*p* < 0.01, ∗*p* < 0.05. (A), (D), and (I) show examples of BRET data. Data shows mean ± SEM.

Cinacalcet also increased BRET between CaSR and Venus-Rab4 in a dose-dependent manner and increased pEC50 values, indicating that the PAM increases receptor recruitment to the early endosome-to-recycling endosome pathway (Figures 9A–9C). NPS-2143 reduced BRET between CaSR and Rab4, consistent with reduced receptor trafficking (Figures 9D and 9E). Pre-incubation of cells with increasing concentrations of cinacalcet also increased recruitment of Nluc-Gq to Rab4-containing endosomes, until saturation at high concentrations (Figures 9F and 9G). NPS-2143 reduced ligand-stimulated Gq recruitment (Figure 7H). Thus, allosteric modulators likely facilitate CaSR sustained signaling, in part, by modifying receptor trafficking to Rab4-mediated early endosome-to-recycling pathways. In contrast to Rab4, pre-treatment of HEK-CaSR cells with increasing concentrations of cinacalcet had no effect on BRET responses between CaSR-Nluc and Venus-Rab9, nor did it affect mGq recruitment to Venus-Rab9 membranes (Figures 9J–9M). However, NPS-2143 did impair trafficking of CaSR to Rab9-labeled membranes and increased agonist-induced recruitment of mGq to Rab9 sites (Figures 9N and 9O). Therefore, cinacalcet may preferentially target CaSR to early endosome-recycling pathways to increase sustained signaling.

Previous studies have shown that cinacalcet can enhance CaSR signaling responses in cells expressing AP2σ-R15 mutations and normalize serum calcium in patients with FHH3.^37^ We therefore hypothesized that cinacalcet may improve CaSR signaling in part, by enhancing CaSR sustained signaling. To test this, cells were transfected with the FHH3-associated AP2σ-R15H mutation and exposed to vehicle or 10 nM cinacalcet prior to BRET assessment of mGq recruitment to Venus-tagged membranes. AP2σ-R15H mutations impaired mGq recruitment to Rab5- and Rab4-containing endosomes, while recruitment in cinacalcet-treated cells was not significantly different from AP2σ-WT cells (Figures 9P and 9Q). In contrast, cinacalcet did not restore recruitment of mGq to Rab9 endosomes (Figure 9R). To test whether cinacalcet enhanced endogenous AP2σ-WT or affected the AP2σ-R15H protein, BRET assays were repeated in cells expressing either the AP2σ-R15H plasmid or a siRNA-resistant AP2σ-R15H plasmid. Following AP2σ-siRNA knockdown of endogenous AP2σ-WT, the siRNA-resistant AP2σ-R15H did not enhance mGq recruitment to Rab5 or Rab4 endosomes, indicating that cinacalcet acts on endogenous AP2σ-WT protein (Figures 9S and 9T).

## Discussion

We demonstrate that a dileucine motif within the CaSR cytoplasmic domain is required for AP2-mediated agonist-induced internalization and preferential Gαq/11 spatiotemporal signaling. We provide several pieces of evidence for this mechanism. Firstly, CaSR and AP2σ interact, and this is impaired by mutation of the dileucine motif or AP2σ-R15, consistent with previous studies in AP2σ-R15L mice.^21^ Secondly, examination of CaSR trafficking by TIRF-M and BRET phenocopies that of cells expressing AP2σ-R15 mutants.^10^ Thirdly, the CaSR dileucine and AP2σ-R15 mutants impair trafficking and recruitment of Gq/11 to intracellular vesicles. Finally, CaSR sustained signaling is similarly impaired in cells expressing the CaSR dileucine mutant, AP2σ-R15H mutant, or Dyngo-4a. Our studies demonstrate that the CaSR dileucine motif and AP2σ-R15 mutants provide ideal tools to study CaSR trafficking and sustained signaling and could be useful in elucidating the physiological functions of CaSR in diverse tissues.^11^^,^^12^^,^^13^

Previous studies of CaSR internalization have provided conflicting findings.^10^^,^^23^^,^^24^^,^^25^^,^^29^^,^^38^ These discrepancies are likely due to the wide variety of experimental approaches adopted to investigate internalization. In our studies, we used kinetic approaches to identify multiple CaSR trafficking destinations including early endosomes (Rab5), late endosomes (Rab9), and both the Rab4 and Rab11 recycling endosomes. This suggests that CaSR trafficking is not a homogeneous process that targets the receptor to a single destination but that the receptor can follow multiple pathways, which may have distinct signaling outcomes. Moreover, identification of this diversity in trafficking outcomes potentially unites the findings from previous studies into a coherent, though complex, picture (Figure S14). In the presence of agonist, CaSR internalizes to early endosomes but can then follow different pathways including trafficking via Rab4 or Rab11 to distinct recycling pathways or targeting to Rab9-positive late endosomes and the Golgi. In parallel, receptor activation drives forward trafficking of CaSR via Golgi vesicles to the plasma membrane (Figure 5), consistent with the previously described ADIS mechanism,^10^^,^^23^ which may involve transport from late endosomes to Rab8-positive *trans*-Golgi vesicles, although this remains to be confirmed. Different destinations likely depend upon the protein composition of individual cells (e.g., expression of ubiquitin ligases^24^^,^^39^), receptor phosphorylation patterns, and coupling to β-arrestin, all of which have been shown to influence GPCR and CaSR trafficking.^29^^,^^38^^,^^40^ It is also possible that CaSR internalizes via other endocytic pathways, such as caveolae, which have been described to target CaSR for degradation,^41^ and may not involve AP2 or the CaSR dileucine motif. CaSR undergoes constitutive internalization to Rab5-positive endosomes in the absence of the CaSR dileucine motif, and this pathway may not require clathrin-mediated endocytosis, whereas agonist-driven internalization is impaired when AP2σ-R15 or the CaSR dileucine motif are mutated (Figure 3). Our studies showed that the lysosomal pathway does not appear to be a major destination for internalized CaSR as disruption of lysosomal maturation by bafilomycin had no effect on overall CaSR signaling. Previous studies have indicated that CaSR is trafficked to alternative pathways for degradation, including proteosomes, and agonist may drive the receptor preferentially to these pathways.^10^^,^^23^^,^^29^

Our studies showed agonist-driven recruitment of CaSR and Gq/11 to Rab5-positive early endosomes, while recruitment of mGi, mG12, or mGs to Rab5 was not observed. Moreover, we did not observe recruitment of these three G proteins to other sites in the cell, and treatment of cells with Dyngo-4a did not disrupt signaling by other pathways indicating it is unlikely that these G proteins are able to facilitate CaSR sustained signaling. Furthermore, we showed that disruption of the CaSR dileucine motif that is required for CaSR internalization impaired ligand-induced Gαq/11 recruitment to CaSR but did not affect activation of other G proteins indicating that CaSR preferentially mediates spatiotemporal signaling via Gαq/11. This is consistent with our previous studies in which we showed that sustained signaling was impaired by inhibitors of Gαq/11 or phospholipase C, while the Gαi-inhibitor pertussis toxin had no effect on sustained signaling.^10^ Although the mechanism by which Gq/11 is selectively recruited to intracellular membranes is unknown, recent cryoelectron microscopy studies have shown that CaSR adopts distinct structural conformations when binding to Gαq vs. Gαi.^16^ It is possible that binding of AP2σ to CaSR favors a structural conformation that more readily facilitates coupling to Gαq than to Gαi for sustained signaling. While initial studies investigating GPCR sustained signaling almost exclusively described Gs-mediated signaling,^5^^,^^6^ a growing body of evidence suggests that receptors can couple to Gαq/11 or Gαi/o to mediate these signaling pathways. These include the CLR that activates cytosolic protein kinase C and nuclear ERK by persistent signaling of Gαs and Gαq at endosomes^30^; the NK1R, which activates Gαq in neuronal Rab5 endosomes^31^; the vasopressin type-2 receptor, which utilizes Gαq and Gαs endosomal signaling when activated by arginine-vasopressin^42^; the FFAR2 that couples to Gαi when internalized to facilitate glucagon-like peptide-1 (GLP-1) release from colonic crypts^3^; and ORs that couple to Gαi in neurons.^8^ Moreover, since our initial observation that CaSR selectively engages Gαq/11 to facilitate spatiotemporal signaling, similar selectivity has been shown for other GPCRs including the FFAR2 that signals from endosomes by Gαi, but not Gαq,^3^ and GLP-1 receptor, for which different agonists elicit unique signaling profiles in part by location bias.^43^

CaSR was demonstrated to traffic to Rab4-positive endosomes and Rab4-knockdown impaired signaling. Vesicles containing Rab4 have been described as important sorting points for directing receptors to different pathways, including degradative and recycling pathways distinct from those regulated by Rab11.^44^^,^^45^ Our studies of CaSR recycling suggest that CaSR uses both Rab4 and Rab11 recycling pathways. It is not unusual for GPCRs to use both Rab4 and Rab11 recycling pathways, and such trafficking has been described for AT1R,^46^ β2-adrenergic receptor (β2AR),^47^^,^^48^ NK1R,^49^ and μOR,^40^ some of which also utilize endosomal signals. Phosphorylation patterns have been shown to be important, with non-phosphorylated μOR shown to traffic to the Rab11 pathway and phosphorylated receptor to the Rab4 pathway.^40^ Whether CaSR signaling from Rab4 vs. Rab11 endosomes has distinct physiological outcomes remains to be addressed, although it is possible that signaling involves similar mechanisms to other GPCRs (dopamine-1 receptor,^50^ follicle-stimulating hormone receptor,^1^ luteinizing hormone receptor,^51^ and FFAR2^3^) described to activate acute G protein signaling from early endosomal locations. Our studies of Rab4 knockdown showed that recruitment of CaSR to Rab5-containing endosomes may also be partially disrupted, which would be expected as it is known that Rab4 and Rab5 have some overlap at early endosomes.^52^ Therefore, some of our observations with Rab4 siRNA may be due to a generalized disruption of recruitment to early endosomes. However, it is known that Rab4 is also present at domains distinct from Rab5, and as the siRNA almost completely removed Rab4 protein expression, it is likely that both Rab4^+^Rab5^+^ and Rab4^+^Rab5^−^ endosomes are affected. Further studies of these distinct domains may be required, although disruption of both proteins simultaneously is likely to have a detrimental effect on cell behavior.

In addition to signaling from Rab5 and Rab4, we showed that CaSR traffics to Rab9-positive vesicles, which also recruit mGq. Cannabinoid-1 receptor has been shown to recruit Gαi to Rab9-positive vesicles to mediate signal transduction following receptor internalization.^9^ Moreover, the κ-OR colocalizes with late endosomes and/or lysosomes in an agonist-dependent manner and induces cAMP signaling.^53^ Thus, there is a precedent for signaling from Rab9, and this pathway likely contributes in part to CaSR sustained signaling. Rab9 transport vesicles have been shown to fuse to the *trans*-Golgi network, and previous studies have shown that GPCRs can activate signals from Golgi vesicles by two mechanisms. The first involves a receptor pool localized at the Golgi with pre-associated G proteins,^54^ while other GPCRs are recruited to the Golgi following internalization (i.e., retrograde trafficking) and induce activation of local Gαs and cAMP/protein kinase A signaling.^2^ Although our studies did not show recruitment of mGq to the Golgi, the receptor did colocalize with a Golgi marker, and it is possible that the sustained signaling from Rab9 is able to convey messages to the Golgi regarding Ca^2+^_e_ concentrations to initiate ADIS. The nature of these messages is not yet known, although further studies of signaling patterns (e.g., phosphorylation) in the presence of allosteric modulators that have differential effects on Rab9 signaling could provide critical information on the link between CaSR signaling and ADIS. Some retention of CaSR and Gq at Rab5-positive early endosomes was observed in the presence of Rab9 siRNA at low concentrations of Ca^2+^_e_. However, CaSR signaling was still impaired suggesting that enhanced signaling from Rab5-positive early endosomes is not sufficient to achieve maximal CaSR signaling and that onward trafficking to other vesicles (e.g., Rab9 vesicles) is required.

As CaSR endosomal signaling is reliant on Gαq/11, CaSR sustained signaling is unlikely to occur in tissues in which CaSR is expressed, but Gαq/11 are not. This knowledge could provide insights into the physiological pathways that CaSR endosomal signaling regulates and could explain differences observed in clinical phenotypes between patients with calcium disorders caused by mutations in CaSR and Gα11. Notably, hypercalciuria occurs frequently in patients with autosomal dominant hypocalcemia caused by mutations in CaSR but is rare in those with Gα11 mutations; and conversely, patients with FHH and mice with inactivating Gα11 mutations have no alterations in urinary calcium excretion, compared to patients with inactivating CaSR mutations in which hypocalciuria is common.^55^^,^^56^ This suggests that Gα11 and sustained signaling may not play a major role in the renal handling of calcium. While the presence of hypercalciuria in FHH3 could argue against this, patients with FHH3 generally have an atypical presentation of FHH with marked, frequently symptomatic hypercalcemia and cognitive dysfunction, attributed to the effect of AP2σ-R15 mutations on other membrane proteins.^18^ Within this study, we have shown that AP2σ-R15 mutations do not affect the signaling and trafficking of four GPCRs. However, we did identify a small reduction in signaling by one GPCR, the kisspeptin receptor, that harbors a dileucine motif.^57^ While we do not think AP2σ-R15 mutations affect all receptors with a dileucine motif, as we have previously shown signaling by β2AR, which has a dileucine motif, is not affected by AP2σ-R15 mutations,^10^^,^^58^ it is possible that other membrane receptors may be affected, and this could explain some of the additional symptoms identified in patients with FHH3. Subsequent studies in a diverse range of cell types, or in animal models with AP2σ-R15 mutations,^21^ could provide further information regarding the (patho)physiological effects of CaSR sustained signaling, which may facilitate the design of CaSR-targeting drugs with fewer off-target effects.

We showed that CaSR sustained signaling can be modified by allosteric modulators, and previous studies have shown that cinacalcet and a different NAM, R-568, can affect CaSR endocytosis.^29^ Although the mechanism is unknown, it is possible that the allosteric modulators lock the CaSR in a conformation that more readily favors interaction with other proteins required for sustained signaling, as was hypothesized for the differential effects of opioid drugs on the κ-OR.^8^ We also observed that cinacalcet enhanced sustained signaling by the Rab5-Rab4 pathway but did not affect recruitment of G protein to Rab9. Cinacalcet has previously been observed to preferentially modulate Ca^2+^_i_, compared to ERK signaling, and to enhance membrane ruffling,^59^ and it is possible that these differences could be attributed to sustained vs. plasma membrane signaling. The observation of biased signaling by cinacalcet provides evidence that CaSR allosteric modulators could be designed to selectively activate sustained or plasma membrane signaling pathways to provide distinct physiological outcomes. This is important for GPCRs, such as CaSR, that are widely expressed in diverse tissues, couple promiscuously to G proteins, and bind a range of ligands. Moreover, the utility of cinacalcet, which is approved for use in some cases of hyperparathyroidism, is limited by adverse gastrointestinal effects that can reduce patient compliance.^26^ Further studies of how cinacalcet mediates sustained signaling in different tissues, including the gastrointestinal tract where CaSR is expressed,^60^ could provide further insights into how these side effects arise and aid drug design to avoid these effects. The ability of drugs to selectively modulate endosomal signaling has been demonstrated for several GPCR compounds, including lipid-conjugated antagonists that accumulate in endosomes to block sustained signaling by CLR and NK1R and consequently reduce neuronal excitation and prolong antinociceptive actions,^30^^,^^31^ and low-molecular-weight agonistic compounds for the follicle-stimulating hormone receptor that exhibit enhanced endosomal signaling and recycling due to a differential sensitivity to APPL1 regulation.^1^

In summary, we have demonstrated that a CaSR endocytic motif is required to activate spatially distinct sustained signaling pathways involving Rab5, Rab4, and Rab9 and that allosteric modulators can modify sustained signaling in a biased manner. These studies could help inform the design of future allosteric modulators that preferentially activate sustained signaling pathways to improve tissue specificity and reduce off-target effects of hyperparathyroidism treatments.

### Limitations of the study

There are a number of limitations to these studies including that we used Rab proteins as markers of compartments. As the expression of these Rab proteins is not exclusively restricted to these intracellular compartments, additional markers may need to be investigated in future studies to strengthen the conclusions. Additionally, these studies rely on overexpression in cell lines as there are no physiologically relevant model cell lines (i.e., of parathyroid) that endogenously express CaSR and recapitulate the behavior of the tissue of interest.

## Resource availability

### Lead contact

Further information and requests for resources and reagents should be directed to and will be fulfilled by the lead contact, Caroline M. Gorvin (c.gorvin@bham.ac.uk).

### Materials availability

Details of the sources of all plasmids are described within this manuscript. All plasmid constructs generated for the manuscript will be made available upon request. Plasmid constructs obtained from other researchers are detailed within the key resources table. Material transfer agreements may be required for some constructs from other researchers.

### Data and code availability

• Original western blot images have been deposited at the open science framework: https://osf.io/7scdk/. All uncropped images have been deposited at the open science framework: https://osf.io/2whtj/. Microscopy data reported in this paper will be shared by the lead contact upon request.
• All original code has been deposited at the open science framework https://osf.io/8uy37/.

## Acknowledgments

This study received funding from an 10.13039/501100000691Academy of Medical Sciences Springboard Award supported by the 10.13039/501100000274British Heart Foundation, 10.13039/501100000361Diabetes UK, the 10.13039/501100022370Global Challenges Research Fund, the Government Department of Business, Energy and Industrial Strategy and the 10.13039/100010269Wellcome Trust, Ref: SBF004|1034 (C.M.G.), and a Sir Henry Dale Fellowship jointly funded by the Wellcome Trust and the Royal Society, grant no. 224155/Z/21/Z (C.M.G.). The authors gratefully acknowledge Joao Correia and Shannon O’Brien at the University of Birmingham for assistance in demonstrating microscope setup.

## Author contributions

Conceptualization, C.M.G.; methodology, M.T.G., C.J.M., and C.M.G.; investigation, R.A.W. and C.M.G.; resources, L.Z., C.J.M., and C.M.G.; software, M.T.G.; formal analysis, M.T.G. and C.M.G.; writing – original draft, M.T.G., C.J.M., and C.M.G.; writing – review and editing, R.A.W., M.T.G., L.Z., C.J.M., and C.M.G.

## Declaration of interests

The authors declare no competing interests.

## STAR★Methods

### Key resources table

**Table:** 

REAGENT or RESOURCE	SOURCE	IDENTIFIER
**Antibodies**		

Rabbit polyclonal anti phospho-44/42 MAPK (pERK1/2)	Cell Signaling Technologies	Catalog. no. 9101L; RRID:AB_331646
Rabbit polyclonal anti total-44/42 MAPK (Total ERK1/2)	Cell Signaling Technologies	Catalog. no. 4695S; RRID:AB_390779
Mouse monoclonal anti-CaSR, clone ADD	Abcam	Catalog. no. ab19347 (5C10); RRID:AB_444867
Rabbit polyclonal anti-calnexin	Millipore	Catalog. no. AB2301; RRID:AB_10948000
Mouse monoclonal anti-FLAG M2 antibody	Sigma	Catalog. no. F3165; RRID:AB_259529
Alexa Fluor 647 donkey anti-mouse secondary antibody	Abcam	Catalog. no. ab181292; RRID:AB_3351687
Rabbit polyclonal anti-HA biotinylated	Abcam	Catalog. no. ab26228; RRID:AB_2115899
Anti-mouse IgG (H+L), F(ab')2 Fragment (Alexa Fluor® 647 Conjugate)	Cell Signaling Technology	Catalog. no. 4410; RRID:AB_1904023
Anti-rabbit IgG (H+L), F(ab')_2_ Fragment (Alexa Fluor® 488 Conjugate)	Cell Signaling Technology	Catalog. no. 4412; RRID:AB_1904025
Rabbit polyclonal anti-TGN38 antibody	Novus	Catalog. no. NBP1-03495; RRID:AB_1522533
Goat Anti-Mouse IgG HRP Conjugated	Bio-Rad	Catalog. no. 1706516; RRID AB_2921252
Donkey Anti-Rabbit IgG, HRP Conjugated	Cytiva	Catalog. no. NA934; RRID:AB_772206
Mouse monoclonal anti-GFP	Merck	Catalog. no. MAB131890; RRID: AB_3665666
Mouse monoclonal anti-HA	BioLegend	Catalog. no. 901514, RRID:AB_2565336

**Chemicals, peptides, and recombinant proteins**		

Dyngo-4a	Abcam	Catalog. no. ab120689; CAS no. 1256493-34-1
Brefeldin-A	Merck	Catalog. no. B7651-5MG; CAS no. 20350-15-6
Cycloheximide	Sigma	Catalog. no. C7698-1G; CAS no. 66-81-9
Bafilomycin-A1	Tocris	Catalog. no. 1334; CAS no. 88899-55-2
Cinacalcet	Cambridge Bioscience	Catalog. no. CAY16042-10 mg; CAS no. 364782-34-3
NPS-2143 hydrochloride	Abcam	Catalog. no. ab145050; CAS no. 324523-20-8
GIP (human)	BioTechne	Catalog. no. 2084; CAS no. 100040-31-1
Glucagon-like peptide 1 (7-36) amide (human)	BioTechne	Catalog. no. 2082; CAS no. 107444-51-9
NDP-MSH	BioTechne	Catalog. no. 3013; CAS no. 75921-69-6
Somatostatin (SST-14)	Sigma	Catalog. no. S9129-5MG; CAS no. 38916-34-6
Ghrelin	Sigma	Catalog. no. 494126; CAS no. 304853-26-7
Isoproterenol	Sigma	Catalog. no. 420355; CAS no. 51-30-9
Pertussis toxin	Sigma	Catalog. no. P2980; CAS no. 70323-44-3
[D-Ala2, D-Leu5]-enkephalin (DADLE)	Abcam	Catalog. no. ab120673; CAS no. 63631-40-3
SNAP-Surface Alexa Fluor 647	NEB	Cat#S9136S

**Critical commercial assays**		

Nano-Glo® Luciferase Assay System	Promega	Catalog. no. N1150
Dual-Glo® Luciferase Assay System	Promega	Catalog. no. E2940
GloSensor cAMP reagent	Promega	Catalog. no. E1291
HTRF IP-One Gq Detection Kit	Revvity	Catalog. no. 62IPAPEB

**Experimental models: Cell lines**		

Adherent HEK293	Agilent	RRID:CVCL_9804; Catalog. no. 240085

**Oligonucleotides**		

Rab4 (RAB4A) Human siRNA Oligo Duplex (Locus ID 5867)	Origene	Catalog. no. SR303946
RAB9B Human siRNA Oligo Duplex (Locus ID 51209)	Origene	Catalog. no. SR309623
AP-2σ1 siRNA (human) Entrez Gene ID: 1175	SantaCruz Biotechnologies	Catalog. no. sc-97710

**Recombinant DNA**		

cAMP Glosensor-22F	Promega	Catalog. no. E2301
pRL-null	Promega	GenBank® Accession Number AF025844
pGL4.33[luc2P/SRE/Hygro]	Promega	Catalog. no. E1340
LgBiT-C-CaSR-WT	This manuscript	N/A
SmBiT-C-CaSR-WT	This manuscript	N/A
LgBiT-N-AP2σ-WT	Chris McCabe, University of Birmingham^61^	N/A
SmBiT-N-AP2σ-WT	Chris McCabe, University of Birmingham^61^	N/A
LgBiT-N-AP2σ-R15H	This manuscript	N/A
SmBiT-N-AP2σ-R15H (*AP2S1* R15H FHH3 mutation)	This manuscript	N/A
SmBiT-C-CaSR-AA (*CASR* LL1013-1014AA mutation)	This manuscript	N/A
SmBiT-C-CaSR-AllA (*CASR* 1009-1014 to alanine)	This manuscript	N/A
SmBiT-C-CaSR-R>A (*CASR* R1009A mutation)	This manuscript	N/A
SmBiT-C-CaSR-H>A (*CASR* H1010A mutation)	This manuscript	N/A
SmBiT-C-CaSR-P>A (*CASR* P1012A mutation)	This manuscript	N/A
SmBiT-C-CaSR-978A (*CASR* S978A mutation)	This manuscript	N/A
SmBiT-C-CaSR-1004A (*CASR* S1004A mutation)	This manuscript	N/A
SmBiT-C-CaSR-1006A (*CASR* T1006A mutation)	This manuscript	N/A
SmBiT-C-CaSR-1008A (*CASR* T1008A mutation)	This manuscript	N/A
pBiT1.1 C [TK/LgBiT]	Promega	Catalog. no. #N196A
LgBiT-Gα11	Asuka Inoue, Tohoku University^62^	N/A
LgBiT-Gαi1	Asuka Inoue, Tohoku University^62^	N/A
BSEP-CaSR-WT	Gerda Breitwieser, Florida State University^23^	N/A
BSEP-CaSR-AA (LL1013-1014AA mutation)	This manuscript	N/A
Venus-Kras	Nevin Lambert, Augusta University^35^	N/A
Venus-Rab1	Kevin Pfleger, University of Western Australia^63^	N/A
Venus-Rab4	Kevin Pfleger, University of Western Australia^63^	N/A
Venus-Rab5	Nevin Lambert, Augusta University^35^	N/A
Venus-Rab8	Kevin Pfleger, University of Western Australia^63^	N/A
Venus-Rab9	Kevin Pfleger, University of Western Australia^63^	N/A
pCMV-HA-Rab11	This manuscript	N/A
Venus-MoA	Nevin Lambert, Augusta University^35^	N/A
Venus-ABC	Nevin Lambert, Augusta University^35^	N/A
Venus-mG12	Nevin Lambert, Augusta University^35^	N/A
Venus-mGi	Nevin Lambert, Augusta University^35^	N/A
Venus-mGq	Nevin Lambert, Augusta University^35^	N/A
Venus-mGs	Nevin Lambert, Augusta University^35^	N/A
Nluc-mG12	Nevin Lambert, Augusta University^35^	N/A
Nluc-mGi	Nevin Lambert, Augusta University^35^	N/A
Nluc-mGq	Nevin Lambert, Augusta University^35^	N/A
Nluc-mGs	Nevin Lambert, Augusta University^35^	N/A
Nluc-CaSR-WT	This manuscript	N/A
Nluc-CaSR-AA (LL1013-1014AA mutation)	This manuscript	N/A
pcDNA3.1-CaSR-WT	Caroline Gorvin, University of Birmingham (Lead author)^15^	N/A
pcDNA3.1-CaSR-AA (LL1013-1014AA mutation)	This manuscript	N/A
pcDNA3.1-CaSR-AllA	This manuscript	N/A
pcDNA3.1-FLAG-CaSR-WT	This manuscript	N/A
FLAG-SNAP-CaSR (Rat)	Hans Bräuner Osborne, University of Copenhagen^29^	N/A
pIRES-puro AP2σ-WT (WT *AP2S1* sequence)	This manuscript	N/A
pIRES-puro AP2σ-R15H (*AP2S1* R15H FHH3 mutation)	This manuscript	N/A
LgBiT-IP3R2-SmBiT	Asuka Inoue, Tohoku University^62^	N/A
mCherry-Rab5	Gia Voeltz, University of Colorado^64^	Addgene plasmid ♯49201
pIRES-puro-Gα11	This manuscript	N/A
Venus-1-55-Gγ2	Nevin Lambert, Augusta University^36^^,^^65^	N/A
Venus-156-239-Gβ1	Nevin Lambert, Augusta University^36^^,^^65^	N/A
masGRK3ct-Nluc	Nevin Lambert, Augusta University^36^^,^^65^	N/A
GFP-GNA11	This manuscript	N/A
siRNA-resistant-AP2σ-R15H	Caroline Gorvin, University of Birmingham (Lead author)^10^	N/A
pcDNA3.1-GIPR	Mette Rosenkilde, University of Copenhagen	N/A
pcDNA3.1-GLP-1R	Mette Rosenkilde, University of Copenhagen	N/A
pcDNA3.1-MC3R	This manuscript	N/A
pcDNA3.1-SSTR5	Caroline Gorvin, University of Birmingham (Lead author)^15^	N/A
pcDNA3.1-GHSR	Caroline Gorvin, University of Birmingham (Lead author)	This manuscript
pcDNA-FLAG-β2AR	Robert Lefkowitz, Duke University^66^	Addgene plasmid #14697
OPRD1-Tango (δ-opioid receptor (DOR))	Bryan Roth, University of North Carolina^67^	Addgene plasmid #66461
HA-SNAP-GHSR	Caroline Gorvin, University of Birmingham (Lead author)^68^	N/A
HA-SNAP-MC4R	Caroline Gorvin, University of Birmingham (Lead author)^69^	N/A
pcDNA-KISS1R	Andy Babwah, Rutgers University^70^	N/A
MC3R-Rluc8	This manuscript	N/A
SSTR5-Rluc8	This manuscript	N/A
GHSR-Nluc	Caroline Gorvin, University of Birmingham (Lead author)^71^	N/A
pcDNA3.1-HA-Dynamin2-WT	Sandra Schmid, unpublished	Addgene plasmid #34684
pcDNA3.1-HA-Dynamin2-K44A	Sandra Schmid, unpublished	Addgene plasmid #34685
GFP-Rab11-DN	Richard Pagano^72^	Addgene plasmid #12678

**Software and algorithms**		

Custom code (HILO analysis Kras and SNAP-CaSR)	This manuscript	https://osf.io/8uy37/
ImageJ	Schneider et al., 2012^73^	RRID:SCR_003070
Microsoft Excel	Microsoft Office	RRID:SCR_016137
GraphPad Prism	GraphPad Prism	RRID:SCR_002798
NIS-Elements	NIS-Elements	RRID:SCR_014329
Inkscape	Inkscape	RRID:SCR_014479

### Experimental model and study participant details

#### Cell-lines

Adherent HEK293 (AdHEK) cells were purchased from Agilent Technologies. AdHEK cells were maintained in DMEM-Glutamax media (Merck) with 10% fetal bovine serum (Merck) at 37°C, 5% CO_2_. HEK293 cells originated from a human female fetus. The cell-line was not formally authenticated but was purchased from a reputable source, cells appeared morphologically similar to AdHEK cells and cells were not maintained for longer than fifty passages.

Generation of the AdHEK CaSR stable cell-line (HEK-CaSR) has been described.^15^ HEK-CaSR cells were maintained in DMEM-Glutamax media (Merck) with 10% fetal bovine serum (Merck) and 500 μg/mL geneticin at 37°C, 5% CO_2_.

AdHEK cell-lines stably expressing either AP2σ-wild-type or AP2σ-R15H were generated using a pIRES-puro-AP2σ plasmid. Cells were plated in 6-well plates, transfected with 1000ng plasmid, then puromycin selection media added 48-hours later. AP2σ expression was assessed by RT-PCR. RNA was extracted from cells using the rNeasy kit (Qiagen) and first-strand cDNA generated using a Quantitect reverse transcription kit (Qiagen). Cells were maintained in DMEM-Glutamax media (Merck) with 10% fetal bovine serum (Merck) and 10 μg/mL puromycin at 37°C, 5% CO_2_.

All cells were routinely screened for mycoplasma infection using the TransDetect Luciferase Mycoplasma Detection kit (Generon).

Expression constructs were transiently transfected into AdHEK using Lipofectamine 2000 (LifeTechnologies), following manufacturer’s instructions.

### Method details

#### Plasmid constructs and compounds

A full list of plasmids with their source can be found in the key resources table. Mutations were introduced into plasmids by site-directed mutagenesis using the Quikchange Lightning Kit (Agilent Technologies) with oligonucleotides obtained from Merck. Dyngo-4a (Abcam) was used at a concentration of 30μM and Brefeldin-A (Merck) at 5μg/ml, with cells pre-incubated for 30 minutes prior to experiments. Cycloheximide (Sigma) was used at a concentration of 50μg/ml, with cells pre-incubated for four hours prior to experiments. Bafilomycin-A1 was purchased from Tocris and used at a concentration of 100nM, with cells pre-incubated for one hour prior to experiments. Cinacalcet (Cambridge Bioscience) was used at 10nM or at a concentration range (5-100nM) and NPS-2143 (Abcam) at 20nM, for 1 hour prior to experiments. GIP, GLP-1 and NDP-MSH were purchased from BioTechne, SST-14 and ghrelin were purchased from Sigma, NDP-MSH was purchased from Cambridge Bioscience, [D-Ala2, D-Leu5]-enkephalin (DADLE) was purchased from Abcam, Isoproterenol was purchased from Sigma. KP-54 was a gift from Professor Waljit Dhillo, Imperial College London. siRNAs directed against Rab4A (SR303946), Rab9B (SR309623) or scrambled sequences were purchased from Origene and those directed against AP2σ purchased from SantaCruz Biotechnologies (sc-97710). All experiments were performed with a cocktail of three different siRNAs of Rab4 or Rab9.

#### NanoBiT assays

NanoBiT assays were performed using methods adapted from previous studies.^74^ NanoBiT constructs were purchased from Promega and CaSR cloned into the LgBiT-C and SmBiT-C plasmids and AP2σ cloned into all four NanoBiT plasmids.^61^ Cells were plated in 6-well plates and transfected 24-hours later with 250ng LgBiT and SmBiT plasmids. Following 48-hours, cells were harvested in FluoroBrite DMEM phenol red-free complete media that contains ∼2mM calcium (with 10% FBS and 2mM L-Glutamine, Life Technologies) seeded across 8 wells of a white 96-well plate at 40,000 cells/well in 40μl media. Approximately 4 hours later, each well was loaded with 40μL NanoLuc substrate (Promega) and luminescence baseline signal read on a Pherastar FSX (BMGLabtech) or a Glomax (Promega) plate reader at 37°C. Data was normalized to luminescence values in the negative control (CaSR-SmC and LgC-Empty). For assays with agonist, cells were incubated with Ca^2+^ and Mg^2+^-free Hank’s buffered saline solution (HBSS) for at least 30 minutes, then four baseline recordings made, followed by agonist addition at 8 minutes and recordings made for a further ∼50 minutes. Plates were read at 37°C. NanoBiT dissociation assays were performed as previously described^15^ with 250ng SmC-CaSR and 250ng LgC-Gα11, Gαi1. Statistical analyses were performed using Graphpad Prism 7.

#### Western blot analysis

Western blot analysis was performed to assess expression of transfected CaSR, detect phosphorylated ERK1/2 protein and measure siRNA knockdown. For CaSR expression studies, either pcDNA3.1-CaSR-WT and CaSR-AllA, or BSEP-CaSR-WT and BSEP-CaSR-AA were transfected at 1μg per well in a 6-well plate. Cells were lysed 48-hours later in NP40 buffer and western blot analysis performed as described.^10^ Blots were blocked in 5% marvel/TBS-T, then probed with anti-CaSR (mouse monoclonal, Catalog. number 5C10, ADD, Abcam) and anti-calnexin (rabbit polyclonal, Catalog. number AB2301, Millipore) antibodies. For testing the effects of CaSR-Nluc on CaSR-mediated generation of pERK1/2, cells were transfected with 1μg plasmid and 36 hours later media was replaced with Ca^2+^ and Mg^2+^-free HBSS. At least 30 minutes later, cells were stimulated with 3mM Ca^2+^ for 5 minutes, then cells lysed and western blot performed. For sustained signaling studies, all media was replaced with Ca^2+^ and Mg^2+^-free HBSS and incubated for at least 30 minutes, then cells were stimulated with 5mM CaCl_2_ for 5 minutes, followed by incubation in media containing 0.1mM CaCl_2_ for 0-60 minutes prior to lysis. After analysis for pERK1/2 (rabbit polyclonal, Catalog. number 9101L, Cell Signaling Technologies) in bovine serum albumin (BSA)/TBS-t, blots were stripped and re-probed with an anti-total ERK1/2 antibody (rabbit polyclonal, Catalog. number 4695S, Cell Signaling Technologies). For tests of siRNA knockdown, cells in 6-well plates were transfected with 500ng per well of i) Venus-Rab4 and Scrambled siRNA, ii) 500ng Venus-Rab4 and Rab4 siRNA, iii) 500ng Venus-Rab9 and Scrambled siRNA, or iv) 500ng Venus-Rab9 and Rab9 siRNA. Blots were probed with anti-Venus (1:500, mouse monoclonal, Catalog. number MAB131890, Merck), then stripped and re-probed with anti-calnexin (rabbit polyclonal, Catalog. number AB2301, Millipore). For all blots either the Goat Anti-Mouse IgG HRP Conjugated (Catalog. number 1706516, Bio-Rad) or Donkey Anti-Rabbit IgG, HRP Conjugated (Catalog. number NA934, Cytiva) secondary antibodies were used at a 1:3000 dilution. Blots were visualized using the Immuno-Star WesternC kit (BioRad) on a BioRad Chemidoc XRS+ system. Densitometry was performed using ImageJ (NIH), and protein quantities normalized to calnexin (CaSR studies) or total ERK1/2 (p-ERK1/2 studies).

#### ELISA

Cells were plated onto coverslips in 6-well plates and transfected with 1μg of pcDNA3.1-CaSR-WT, CaSR-AA or CaSR-AllA, or FLAG-, Nluc-, LgC- or SmC-CaSR per well. Cells were replated in 96-well plates 48-hours after transfection to assess expression of cell surface CaSR. Cells were left to settle for at least four hours, then fixed in 4% paraformaldehyde/PBS (Sigma). Following blocking in serum, cells were incubated with an anti-CaSR antibody (1:500, mouse monoclonal, Catalog. number 5C10, ADD) for 3 hours at room temperature, then an alkaline phosphatase conjugated secondary antibody for an hour. Plates were washed in PBS-t, then para-nitrophenyl phosphate (pNPP, ThermoFisher) added as a substrate for alkaline phosphatase, and absorbance read at 405nm on a Glomax plate reader at room temperature. Background fluorescence was subtracted from all readings, then data expressed relative to mock transfected cells.

#### Total internal reflection fluorescence microscopy (TIRF-M)

Cells were transfected with BSEP-CaSR-WT or BSEP-CaSR-AA,^23^ 24-hours prior to recordings and TIRF-M performed as described.^10^^,^^75^ Images were obtained with a customized Olympus IX-81 TIRF microscope with a 60x/1.45 Apo lens (Olympus). The 488nm line of an argon ion laser (Melles Griot) was used to excite SEP-CaSR, and a 561nm line of a steady-state diode laser used to excite BTx-594. Two excitation ports with separate focal pathways were used to make independent laser adjustments. The emission pathway for both fluorescent reporters was imaged simultaneously with an image splitter (Dual View, Optical Insights) generating side-by-side images of both emission wavelengths on the chip of an electron multiplying charge-coupled device (EMCCD) camera (Cascade II 512B, Roper Scientific). Alignment of the two emission channels was corrected using 200nm tetraspeck beads (Molecular Probes). Cells were imaged in osmotically balanced extracellular medium (140mM NaCl, 5mM KCl, 0.55 MgCl_2_, 10mM HEPES and 10mM D-glucose, pH 7.4) containing appropriate CaCl_2_ concentrations adjusted with NaCl. Cells were placed in this media one hour prior to imaging. Cells were incubated with 5μg/mL BTx-594 (Invitrogen) for 3 minutes prior to imaging. Experiments were performed at 37°C with continuous imaging of cells perfused with basal 0.1mM CaCl_2_ imaging solution for 3 minutes, followed by addition of 3mM CaCl_2_ imaging solution for 10 minutes, then 4 minutes in basal solution. Solutions took 45 seconds from switching the perfusion to reaching the chamber. Images were captured at 10 frames/sec using CellˆR software (Olympus) and analyzed with ImageJ. Fluorescence intensity was measured in each frame of captured movies at both emission wavelengths, and green fluorescence intensity and red fluorescence intensity normalized to the first frame to acquire total surface and internalized CaSR, respectively. Data was analyzed in Graphpad Prism 7.

#### Bioluminescence resonance energy transfer (BRET) assays

NanoBRET assays were performed using methods adapted from previous studies.^35^^,^^36^^,^^65^^,^^71^ For CaSR studies, AdHEK cells were seeded at 100,000 cells/well in 6-well plates and transfected 48-hours later with either 50ng Nluc, 500ng Venus, and 300ng pcDNA3.1-CaSR-WT when required (e.g. in AP2σ cells), or 50ng of each of GRK3-Nluc, Gβ-Venus, Gγ-Venus, pcDNA3.1-CaSR and 100ng pIRES-puro-Gα11. For other GPCRs in AP2σ-WT and AP2σ-R15H, cells were transfected with 500ng Venus and 300ng MC3R-Rluc8, SSTR5-Rluc8 and GHSR-Nluc. For miniG control experiments, cells were transfected with either 300ng pcDNA-FLAG-β2AR, 500ng Venus-Rab5 and 50ng Nluc-mGs or 300ng OPRD1-Tango, 500ng Venus-Rab5 and 50ng Nluc-mGi. For studies with Dynamin, 300ng of the wild-type or K44A mutant were co-transfected with BRET plasmids. For studies with siRNA-resistant AP2σ-R15H, cells were co-transfected with 100nM *AP2S1* siRNA. Forty-eight hours later, cells were washed with PBS, then detached and resuspended in FluoroBrite complete media and plated across 8-wells in a white 96-well microplate. At least 4 hours later, media was replaced with Ca^2+^- and Mg^2+^-free HBSS (Sigma) and incubated for at least 30 minutes. Nano-Glo reagent (Promega) was then added at a 1:100 dilution for Nluc constructs or Coelenterazine-h (Promega) added at 10μM for Rluc8 constructs and BRET measurements recorded using a PHERAstar or GloMax microplate reader at donor wavelength 475-30 and acceptor wavelength 535-30 at 37°C. The BRET ratio (acceptor/donor) was calculated for each time point. Four baseline recordings were made, then agonist added at 8 minutes and recordings made for a further ∼50 minutes. The average baseline value recorded prior to agonist stimulation was subtracted from the experimental BRET signal. All responses were then normalized to that treated with vehicle to obtain the normalized BRET ratio. AUC was calculated in GraphPad Prism and these values used to plot concentration-response curves with a 4-parameter sigmoidal fit.

#### HILO

AdHEK cells were seeded on 24mm coverslips (VWR) and transfected with 500ng of each plasmid 24-hours prior to experiments. For studies with SNAP-CaSR, SNAP-Surface Alexa Fluor 647 (NEB) was diluted 1:1000 in FluoroBrite complete media and applied to cells for 20-minutes, before washing and imaging. HILO images were acquired on a custom-built TIRF microscope (Cairn Research) comprising an Eclipse Ti2 (Nikon) equipped with an EMCCD camera (iXon Ultra, Andor), a 488 nm diode laser, a hardware Perfect Focus System, a TIRF iLas2 module, and a 100x oil-immersion objective (NA 1.49, Nikon). Coverslips were mounted onto plastic imaging chambers with a rubber seal and filled with imaging medium (Ca^2+^- and Mg^2+^-free HBSS with 10mM HEPES). The objective and samples were maintained at 37°C. Images were acquired on MetaMorph software (Molecular Devices) using a frame exposure of 50–200 ms with two images acquired before ligand stimulation (3mM Ca^2+^_e_) and a subsequent image taken every 30 seconds thereafter, up to 20 minutes. Images were analyzed using ImageJ. Colocalization was measured using the ImageJ plugin JACoP. Regions of interest were selected and cropped so that images contained single cells, then JACoP thresholds for each channel were set using the Costes’ automatic thresholding. JACoP was then used to calculate Pearson’s correlation coefficient for at least 3 regions of interest per cell. Colocalization between an unrelated GPCR (GHSR, growth hormone secretagogue receptor) and intracellular markers in cells exposed to 3mM Ca^2+^_e_ was used as a negative control to determine whether colocalization was unrelated to CaSR function. Additional imaging with SNAP-CaSR, intracellular markers and vehicle was used to compare responses. For analysis of Kras and SNAP-CaSR, a bespoke automated routine was developed in Python to analyse the full timeseries of images (code available at the open science framework https://osf.io/8uy37/.). Briefly, in each image AdHEK cells were segmented using custom-trained models in Cellpose 2.0,^76^ with the resulting segmentation masks then used to extract the variation of pixel intensities across each cell for each frame.

#### Structured illuminated microscopy (SIM)

Cells were plated on 24mm coverslips (VWR) and transfected with 500ng of each plasmid 36-hours prior to experiments. Cells were incubated in Ca^2+^- and Mg^2+^-free HBSS for one hour, then labelled with SNAP-surface Alexa Fluor 647 (NEB) diluted 1:1000 in HBSS with 0.1mM, 3mM, 5mM or 10mM Ca^2+^_e_ (as indicated in figures) for 30 minutes or for studies with antibodies, cells were exposed to Ca^2+^_e_, then fixed, permeabilised and exposed to 1:1000 anti-FLAG M2 mouse monoclonal antibody (F1804, Sigma) or 1:1000 anti-HA rabbit polyclonal (ab26228, Abcam), followed by Alexa Fluor 488 donkey anti-rabbit (Catalog. no. 4412, Cell Signaling Technology) or Alexa Fluor 647 donkey anti-mouse (Catalog. no. 4410, Cell Signaling Technology). For studies with TGN38, cells were exposed to 1:500 anti-TGN38 (NBP1-03495, Novus) with 1:1000 anti-FLAG M2 mouse monoclonal antibody (F1804, Sigma) for CaSR, Alexa Fluor 488 donkey anti-rabbit (Catalog. no. 4412, Cell Signaling Technology) or Alexa Fluor 647 donkey anti-mouse (Catalog. no. 4410, Cell Signaling Technology). For GHSR studies, cells were transfected with 500ng HA-SNAP-GHSR, then labelled with SNAP-surface Alexa Fluor 647 diluted 1:1000 in HBSS with 10nM ghrelin for 30 minutes. Cells were washed 1x in PBS, then fixed in 4% PFA (VWR) in PBS buffer. Imaging was performed in Ca^2+^- and Mg^2+^-free HBSS with 10mM HEPES. Samples were imaged on a Nikon N-SIM system (Ti-2 stand, Cairn TwinCam with 2 × Hamamatsu Flash 4 sCMOS cameras, Nikon laser bed 488 and 647 nm excitation lasers), Nikon 100 × 1.49 NA TIRF Apo oil objective at room temperature. SIM data was reconstructed using NIS-Elements (v. 5.21.03) slice reconstruction. Colocalization was measured using the ImageJ plugin JACoP. Regions of interest were selected and cropped so that images contained single cells, then JACoP thresholds for each channel were set using the Costes’ automatic thresholding. JACoP was then used to calculate Pearson’s correlation coefficient for at least 3 regions of interest per cell.

#### Dual-Glo luciferase reporter assays

Luciferase reporter assays were performed in transiently transfected AdHEK cells as previously described.^10^ Cells were plated in 24-well plates and transiently transfected with 100ng pGL4-SRE luciferase reporter constructs (Promega), 10 ng/ml pRL, and 100ng pcDNA3.1-CaSR-WT or CaSR-AA. Media was changed to serum-free media 12-hours before agonist stimulation. On the day of the experiment, cells were incubated in Ca^2+^ and Mg^2+^-free HBSS for at least 30 minutes, then underwent one of three additions: i) 0.1mM CaCl_2_; ii) 3mM CaCl_2_ for the duration of the experiment (constant); or iii) a 5-minute pulse of 3mM CaCl_2_ followed by 0.1mM CaCl_2_ for the duration of the experiment. Cells were lysed each hour for 12-hours and luciferase assays performed using Dual-Glo Luciferase (Promega), on a Glomax plate reader at room temperature. Luciferase:renilla ratios were expressed as fold-changes relative to responses in vehicle treated cells at time 0.

#### IP_3_ biosensor

AdHEK cells were plated in 6-well plates and transfected 24-hours later with 200ng LgBiT-IP_3_R2-SmBiT plasmid and either 500ng pcDNA3.1-CaSR-WT or pcDNA3.1-CaSR-AA. For siRNA studies, HEK-CaSR cells were transfected with 200ng LgBiT-IP_3_R2-SmBiT and siRNAs. Following 48-hours, cells were harvested in FluoroBrite complete media (ThermoScientific) and seeded in 8 wells of a 96-well plate. Cells were incubated for 4 hours, then all media was replaced with Ca^2+^ and Mg^2+^-free HBSS (Sigma), before loading each well with 40μL NanoGlo substrate (1:100 dilution) and luciferase signal read on a Glomax plate reader at 37°C. Vehicle and agonists were prepared in Ca^2+^ and Mg^2+^-free HBSS at 10x concentration and added to wells following recording of baseline signals for four cycles (equivalent to 8 minutes), then responses read for up to 50 minutes. The average baseline value recorded prior to agonist stimulation was subtracted from the experimental signal, then data normalized to vehicle-treated cells. AUC was calculated in GraphPad Prism and these values used to plot concentration-response curves with a 4-parameter sigmoidal fit.

#### Glosensor cAMP assays

AdHEK or AP2σ-WT and AP2σ-R15H cells were plated in 6-well plates and transfected with 100ng pGloSensor-22F plasmid and either 500ng pcDNA-CaSR-WT, pcDNA-CaSR-AA, or plasmids for GIPR, GLP-1R, MC3R, SSTR5, MC4R (Figures S2 and S4). Forty-eight hours later, cells were seeded in 96-well plates in FluoroBrite complete media (ThermoScientific). Cells were incubated for at least 4 hours, then media changed to 100μL of equilibration media consisting of Ca^2+^- and Mg^2+^-free HBSS containing 2% (v/v) dilution of the GloSensor cAMP Reagent stock solution. Cells were incubated for 2 hours at 37°C. Basal luminescence was read on a Glomax plate reader at 37°C for 8 minutes, then agonist added with 10 μM forskolin (Sigma) for CaSR and SSTR5, or without forskolin for Gs-coupled receptors and plates read for a further 30 minutes. For studies with Dyngo-4a and CaSR, one set of experiments included incubation with forskolin to assess Gi-mediated suppression in cAMP and the other set of experiments used 300 ng/ml pertussis toxin (pre-incubated with cells for six hours) to assess Gs-mediated responses. Data was plotted in GraphPad Prism, area-within the curve calculated and these values used to plot concentration-response curves with a 4-parameter sigmoidal fit.

#### IP-1 assays

IP-1 assays were performed with the Cisbio IP-One Gq HTRF kit (Revvity) in AdHEK cells with 500ng FLAG-CaSR or CaSR-Nluc or in AP2σ-WT and AP2σ-R15H cells transfected with 500ng pcDNA3.1-GHSR or pcDNA-KISS1R. Forty-eight hours later, cells were replated into 384-well plates in Ca^2+^- and Mg^2+^-free HBSS. Agonist dilutions were made in IP-1 stimulation buffer and incubated at 37°C for 30 minutes. Cells were lysed in the kit buffer, assays were performed according to manufacturer’s instructions and as described,^77^ then plates measured on a BMG Labtech PHERAstar at room temperature to measure emission at 620 and 665 nm with excitation at 320nm. The supplied IP-1 standard concentration range were performed in tandem to quantify IP-1 concentrations. Data was normalized to baseline levels and analyzed in GraphPad Prism and these values used to plot concentration-response curves with a 4-parameter sigmoidal fit.

#### Transferrin-uptake assays

Transferrin uptake assays were performed in AdHEK cells using methods adapted from those previously described.^10^^,^^78^ Cells were seeded in black-sided 96-well plates and transfected with scrambled, Rab4 or Rab9 siRNA. Two days later cells were treated with either 5μg/mL transferrin-488 (Molecular Probes), 50μg/mL unlabelled transferrin (Sigma), or combined transferrin-488 and unlabelled transferrin for 30 minutes. Following incubation, cells were washed in PBS and lysed in NP40 buffer. Fluorescence was measured using a Promega Glomax plate reader at 475nm excitation and 525nm emission wavelengths at room temperature. Data was normalized to uptake in untreated cells.

#### Antibody feeding assays

For studies of constitutive vs. agonist-driven internalization of FLAG-CaSR-WT or FLAG-CaSR-AA or studies of internalization with scrambled, Rab4 or Rab9 siRNA and FLAG-CaSR-WT, cells were exposed to 1:1000, cells were plated in 6-well plates with coverslips, then transiently transfected with 200ng receptor 200ng Venus-Rab5 and siRNAs. Two days later cells were incubated in Ca^2+^ and Mg^2+^-free HBSS for at least 30 minutes prior to stimulation. Cells were exposed to Ca^2+^ and Mg^2+^-free HBSS containing 1:1000 FLAG (M2, Sigma) and 3mM Ca^2+^_e_ for 0, 10 or 30 minutes. Cells were then fixed in 4% PFA/PBS, permeabilized with 1% Triton-X100/PBS and incubated with anti-mouse Alexa Fluor 647 (Abcam, ab181292), prior to imaging by SIM.

For plate-based assays of receptor recycling, AdHEK cells were plated in black-walled 96-well plates and for imaging studies of receptor recycling, cells were plated in 6-well plates with coverslips, then transiently transfected with 200ng FLAG-CaSR-WT and scrambled or Rab4 siRNA, or overexpression of Rab11a-WT (100ng) or Rab11a-DN (100ng). Two days later cells were incubated in Ca^2+^ and Mg^2+^-free HBSS for at least 30 minutes prior to stimulation. For recycling studies, cells were then exposed to either Ca^2+^ and Mg^2+^-free HBSS containing: i) no antibody, ii) 1:1000 FLAG (M2, Sigma) antibody with 0mM Ca^2+^_e_ for 30 minutes, iii) 1:1000 FLAG with 3mM Ca^2+^_e_ for 30 minutes, iv) 1:1000 FLAG with 0mM Ca^2+^_e_, followed by acid-strip (0.2M acetic acid, 0.5M NaCl, pH3) and recovery for 3 hours in HBSS, v) 1:1000 FLAG with 3mM Ca^2+^_e_, followed by acid-strip and recovery for 3 hours in HBSS. For imaging studies, some plates were incubated at 4°C to block internalization. Cells were then fixed in 4% PFA/PBS and either immediately immuno-stained (non-permeabilized) or permeabilized with 1% Triton-X100/PBS. Cells were incubated with anti-mouse Alexa Fluor 647 (Abcam, ab181292) and either fluorescence quantified on a Glomax plate reader (excitation 627nm and emission 660-720 filter) measured at room temperature or imaged by SIM.

#### pERK1/2 AlphaScreen assays

AlphaScreen assays (PerkinElmer) were performed in 48-well plates in AdHEK cells transiently transfected with 200ng pcDNA3.1-CaSR-WT and scrambled, Rab4 or Rab9 siRNA. Two days later cells were incubated in Ca^2+^ and Mg^2+^-free HBSS for at least 30 minutes prior to a 5-minute treatment with 3mM Ca^2+^_e_, followed by incubation in HBSS containing 0.1mM CaCl_2_ for 0-60 minutes prior to lysis in Surefire buffer and pERK1/2 (Catalog. no. TGRESB500, PerkinElmer) and total ERK1/2 (Catalog. no. TGRTESB500, PerkinElmer) assays performed according to manufacturer’s instructions. Assays were measured at room temperature on a PheraStar FS microplate reader and pERK1/2 normalized to total ERK1/2.

### Quantification and statistical analysis

Data was plotted and statistical analyses performed in Graphpad Prism 7. Normality tests (Shapiro-Wilk or D’Agostino-Pearson) were performed on all datasets to determine whether parametric or non-parametric statistical tests were appropriate. Statistical analyses were performed as indicated in figure legends. The number of independent biological replicates is denoted by N. A p value of <0.05 was considered statistically significant. In all figures, statistical significance is indicated by asterisks as ∗∗∗∗p < 0.0001, ∗∗∗p < 0.001, ∗∗p < 0.01, ∗p < 0.05. All raw immunoblots are available at the open science framework: https://osf.io/7scdk/.
