# Supplementary material for: A calcium-sensing receptor dileucine motif directs internalization to spatially distinct endosomal signaling pathways
Source: iScience. 2025 May 13;28(6):112651. doi: 10.1016/j.isci.2025.112651 (PMC12159506; doi:10.1016/j.isci.2025.112651)
Supplement: Document S1. Figures S1–S12 and Table S1 [file mmc1.pdf]

iScience, Volume *28*

Supplemental information

A calcium-sensing receptor dileucine motif directs internalization to spatially distinct endosomal signaling pathways

Rachael A. Wyatt, Meurig T. Gallagher, Ling Zha, Christopher J. McCabe, and Caroline M. Gorvin

Table S1 **Prediction of CaSR endocytic motifs, related to Figure 1**

Type of Endocytic Motif	Residues	Sequence	Structure location
Dileucine	169-174	SSSRLL	ECD
	275-280	DLEPLI	ECD
	1009-1014	RHEPLL	C-terminus
Tyrosine-based	95-98	YRIF	ECD
	310-313	YFHV	ECD
	411-414	YTHL	ECD
	421-424	YLAV	ECD
	435-438	YTCL	ECD
	489-492	YSII	ECD
	510-513	YYNV	ECD
	829-832	YGKF	ECL3

Location of endocytic motifs in CaSR predicted from the Eukaryotic Linear Motif (ELM) prediction tool (http://elm.eu.org/search.html). The locations of the motifs were derived from a published CaSR cryo-EM structure [Ref S1]. Only the -RHEPLL- motif is feasible as an internalization sequence as the other putative motifs are located in the extracellular region of the receptor.

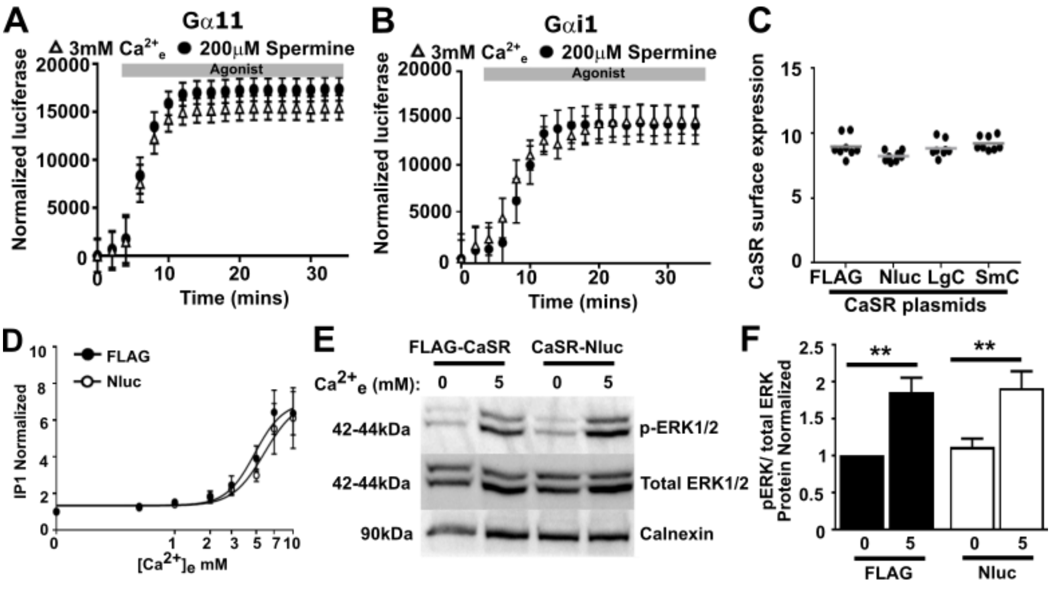

Figure S1 NanoBiT and CaSR-Nluc plasmids signal and express normally, related to Figure 1 and 2

G protein activation measured by NanoBiT association assays between SmBiT-C-CaSR and (**A**) LgBiT-Gα11 or (**B**) Gαi1. Values were normalized to vehicle responses in N=4. (**C**) CaSR cell surface expression of FLAG, Nluc, LgC and SmC constructs measured by ELISA in N=8. Data is expressed relative to mock-transfected cells. (**D**) IP-1 responses of cells expressing FLAG-CaSR or CaSR-Nluc constructs. N=7. (**E**) Western blot analyses of p-ERK1/2 responses in AdHEK cells expressing FLAG-CaSR or CaSR-Nluc. Both constructs had similar responses to 5mM Ca^{2+}_{e}. (**F**) Densitometry showing p-ERK1/2 responses normalized to total ERK1/2 from N=6. Data was analyzed by Kruskal-Wallis one-way ANOVA in C and F, and two-way ANOVA comparing FLAG-CaSR to CaSR-Nluc responses at each [Ca^{2+}_{e}] with Sidak's multiple-comparisons test in D. **p<0.01. Data shows mean±SEM in all panels.

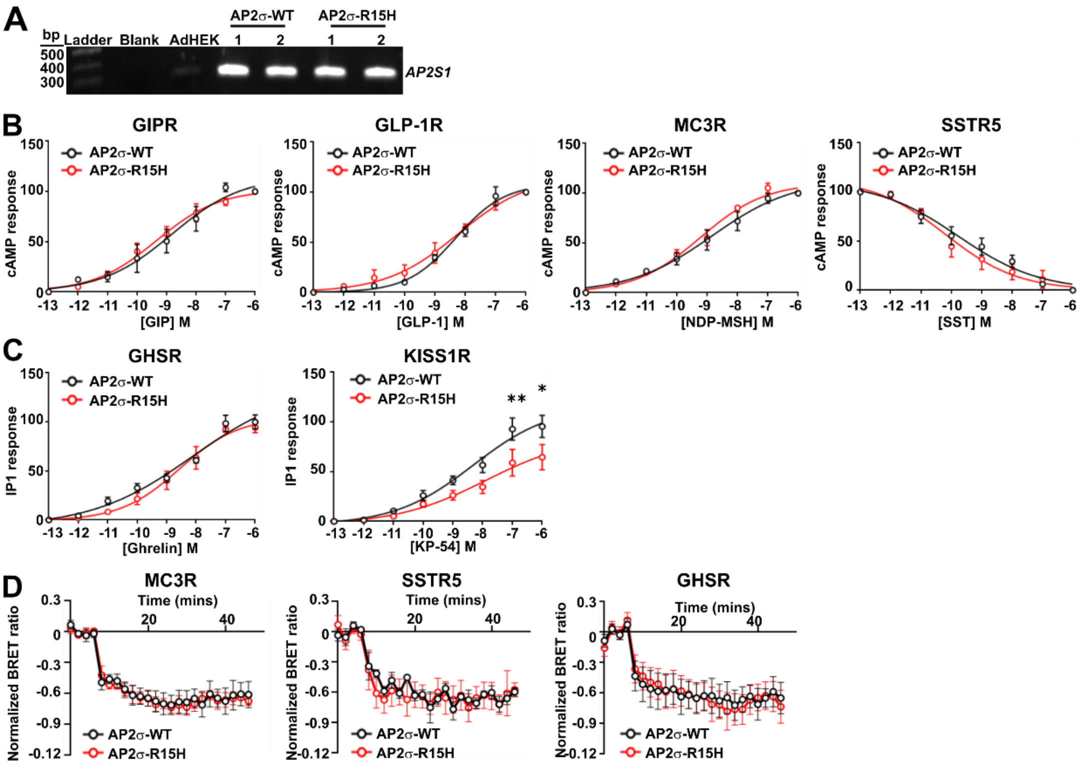

Figure S2 **Generation of AP2σ-WT and AP2σ-R15H stable cell-lines, related to Figure 2**

(**A**) RT-PCR showing enhanced expression of *AP2S1* in HEK293 cells stably overexpressing either AP2σ-WT or AP2σ-R15H. (**B**) Dose-response generated from AUC data in cAMP Glosensor assays of cells expressing HEK-AP2σ-WT or HEK-AP2σ-R15H cells expressing four Gs-coupled GPCRs. N=4 for GIPR and GLP-1R, N=6 for MC3R, N=5 for SSTR5. (**C**) IP-1 responses of HEK-AP2σ-WT or HEK-AP2σ-R15H cells expressing two Gq-coupled GPCRs. N=4 for GHSR and KISS1R. (**D**) BRET ratios measured between Venus-Kras and MC3R-Rluc8, SSTR5-Rluc8 or GHSR-Nluc in HEK-AP2σ-WT or HEK-AP2σ-R15H cells. N=3. Data shows vehicle subtracted responses for each condition. Statistical analyses were performed by two-way ANOVA with Sidak's multiple-comparisons test in B and C and compare AP2σ-WT to AP2σ-R15H at each concentration. All data shows mean+SEM. **p<0.01, *p<0.05.

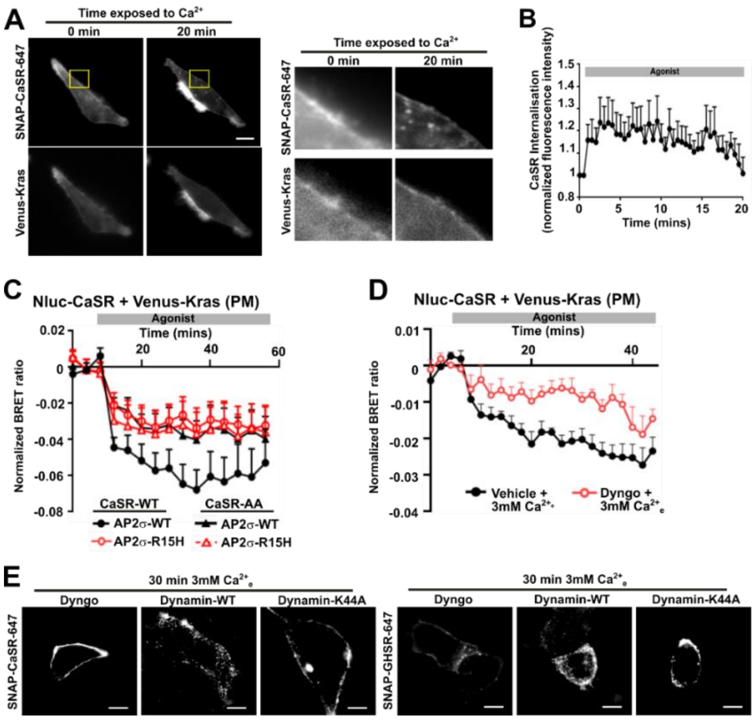

Figure S3 Demonstration that SNAP-CaSR localises to Kras membranes and internalizes normally, related to Figure 2

(**A**) Representative HILO images from HEK293 cells transfected with SNAP-CaSR-WT and Venus-Kras exposed to 3mM Ca^{2+}_{e}, with close-up images shown to the right. Scale, 5µm. (**B**) Quantification of CaSR internalization from images in A, normalized to fluorescence intensity at basal Ca^{2+}_{e}. The amount of cytoplasmic CaSR under basal conditions and following stimulation were quantified. Therefore, the increase in signal corresponds to the increased amount of internalized CaSR. (**C-D**) Example data from Nluc-CaSR and Venus-Kras BRET shown in Figure 2L and Figure 2M. (**E**) Representative SIM microscopy images from cells expressing CaSR or GHSR pre-incubated with Dyngo-4a or overexpressing either dynamin-WT or dynamin-K44A, then exposed to ligand for 30 minutes. Internalization was blocked in cells expressing Dyngo-4a and Dynamin-K44A. Scale, 5µm. All data shows mean+SEM.

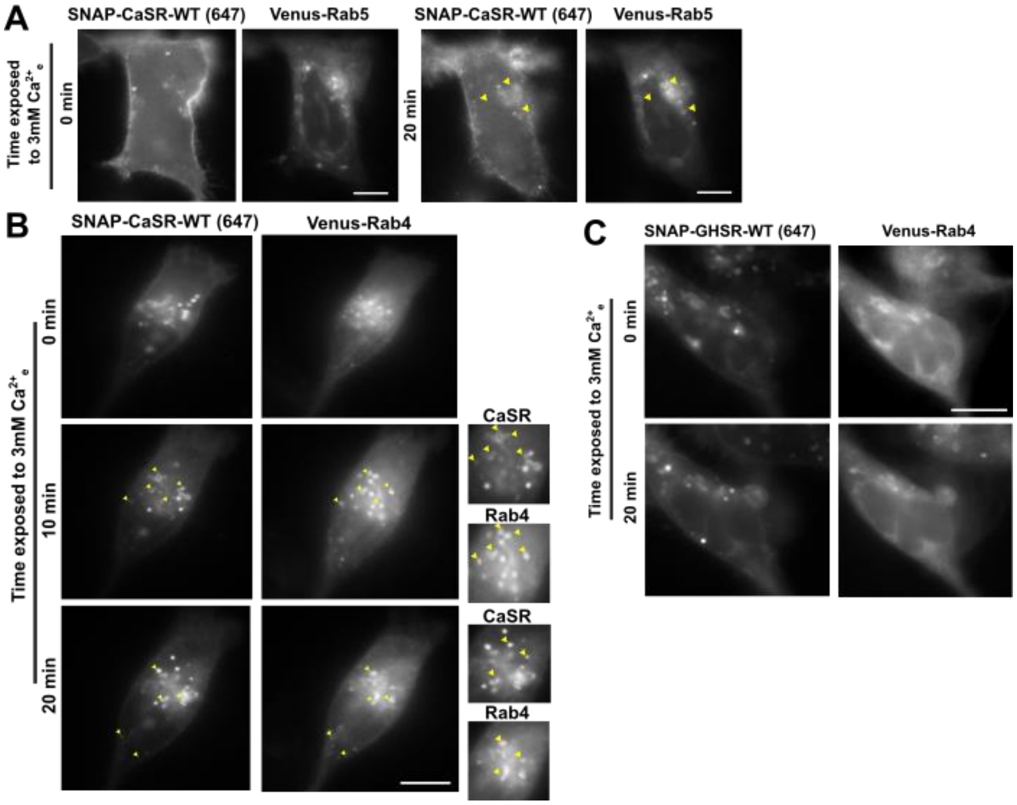

Figure S4 CaSR localizes with Rab5- and Rab4-containing vesicles, related to Figure 3
(**A**) Representative HILO images from cells transfected with SNAP-CaSR-WT and Venus-Rab5. (**B**) Representative HILO images from cells transfected with SNAP-CaSR-WT and Venus-Rab4. Right images show close-up with co-localized vesicles highlighted with arrows Assessment of CaSR and Rab4 in N=11 cells from 3 biological replicates demonstrated a significant increase in colocalization over time (Pearson's coefficient: 0.56±0.02 at basal vs. 0.64±0.03 at 10 mins, $p<0.05$ and 0.70±0.03 at 20 mins, $p<0.01$). (**C**) Representative HILO images from cells transfected with SNAP-GHSR and Venus-Rab4. Scale, 5μm.

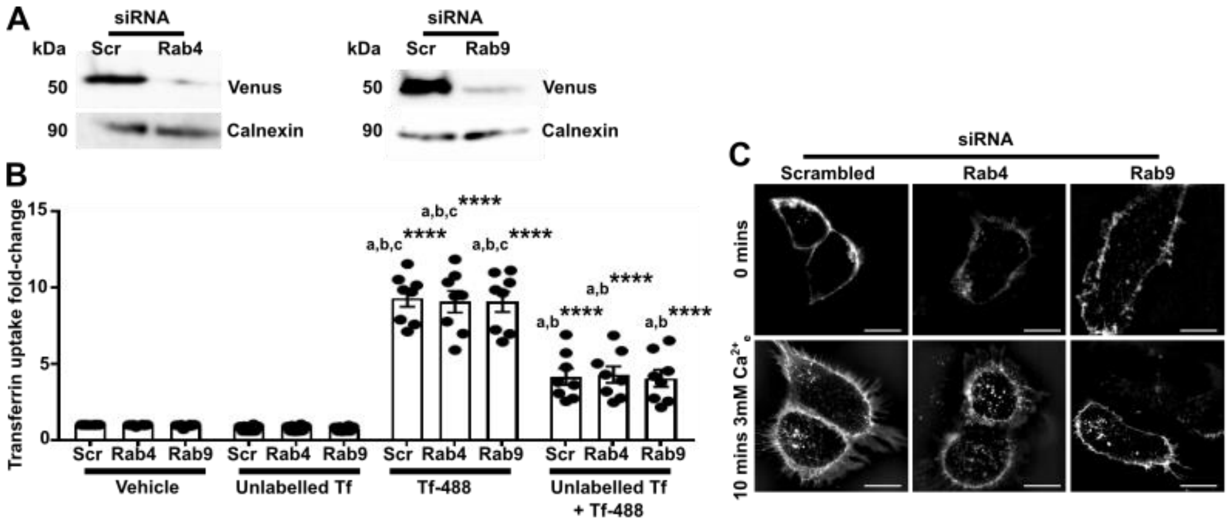

Figure S5 Test of effects of Rab4 and Rab9 siRNAs on CaSR trafficking, related to Figure 4 and 7

(**A**) Western blots showing cells transfected with Venus-Rab4 (left) and Venus-Rab9 (right) and co-transfected with scrambled, Rab4 or Rab9 siRNA. Expression of the Venus protein was used to assess protein knockdown and calnexin was used as a loading control. (**B**) Transferrin (Tf) uptake assays in AdHEK cells exposed to either fluorescent Tf-488 and/or unlabelled Tf. N=8. Data was normalized to responses in vehicle cells. Statistical analyses were performed by one-way ANOVA with Sidak's multiple-comparisons test and show comparisons to vehicle (labelled a), unlabelled Tf (labelled b), unlabelled Tf + Tf-488 (labelled c). There were no significant differences in Tf-488 uptake between scrambled (scr), Rab4 and Rab9 siRNA treated cells. (**C**) Representative SIM images of cells transfected with SNAP-CaSR and scrambled, Rab4 or Rab9 siRNA. Scale, 5μm. Ligand-induced internalisation of SNAP-647 labelled CaSR occurred in all cells. ****$p<0.0001$.

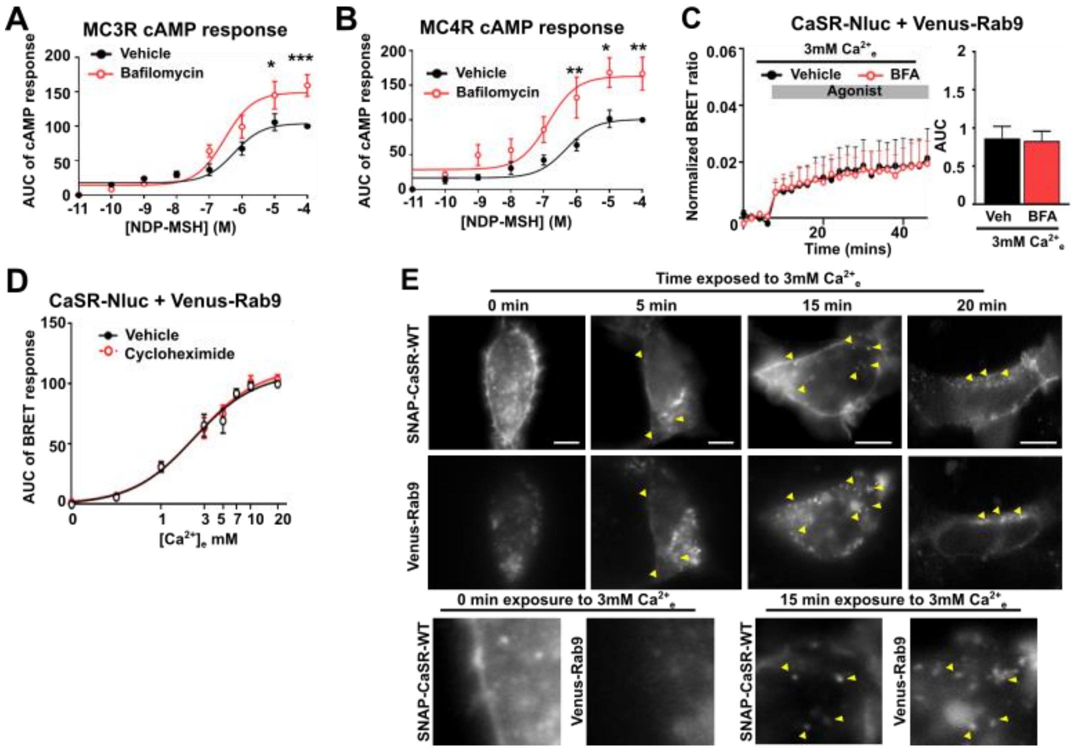

Figure S6 **CaSR trafficking to Rab7 is minimal, while agonist increases CaSR trafficking to the Golgi, related to Figure 5**

Ligand-induced cAMP responses in cells transfected with (**A**) MC3R or (**B**) MC4R and pre-incubated with vehicle or bafilomycin. N=5. (**C**) BRET ratios in cells pre-stimulated with (C) brefeldin-A (BFA) with AUC. N=6. (**D**) Dose-response generated from AUC of ligand-induced BRET between CaSR-Nluc and Venus-Rab9 in cells pre-treated with cycloheximide. N=4. (**E**) HILO images from cells transfected with SNAP-CaSR-WT and Venus-Rab9. Agonist increases co-localization between CaSR and Rab9 (Pearson's coefficient = 0.50±0.02 under basal conditions, 0.61±0.04 at 15 mins, $p<0.02$, and 0.63±0.04 at 20 mins, $p<0.01$). Co-localized vesicles are highlighted with arrows and close-up images shown below. N=14 cells from 4 biological replicates. Scale, 5μm. Data was normalized to vehicle responses in BRET assays. Statistical analyses were performed by two-way ANOVA with Sidak's multiple-comparisons test in A, B, D and unpaired t-test in panel C.

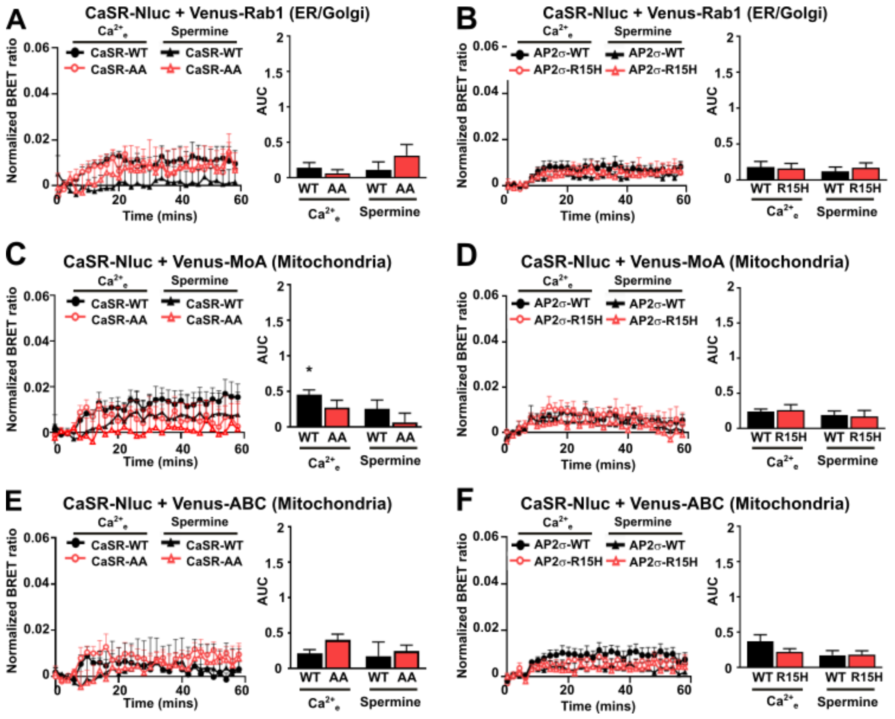

Figure S7 CaSR does not traffic to ER or mitochondrial membranes following internalization, related to Figure 5

(Left) Ligand-induced BRET ratios with AUC measured between: (**A-B**) Rab1-Venus, (**C-D**) MoA-Venus, and (**E-F**) ABC-Venus, with CaSR-WT-Nluc or CaSR-AA-Nluc in HEK293, HEK-AP2σ-WT or HEK-AP2σ-R15H cells. Statistical analyses in black shows vehicle vs. agonist responses. *$p<0.05$. N=6 in panel A, N=8 in B, N=7 in C, D and E, N=8 in F. Statistical analyses were performed by one-way ANOVA with Tukey's multiple-comparisons test. BRET data shows vehicle subtracted responses with mean+SEM.

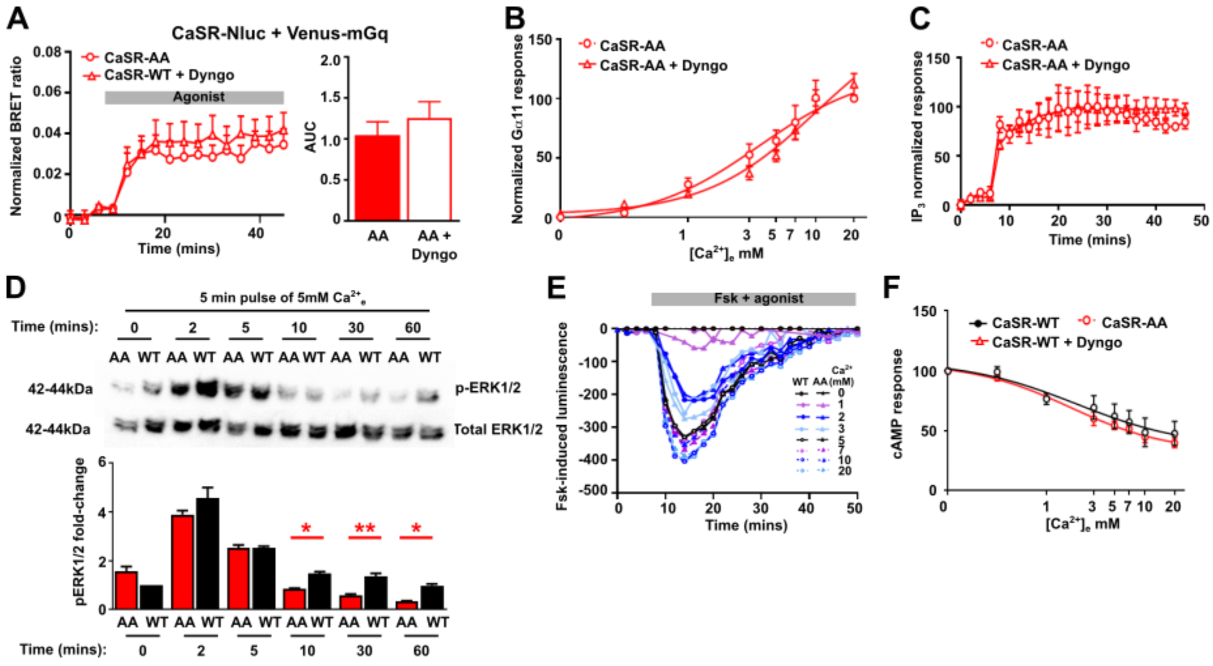

Figure S8 Control experiments for mini-Gs and mini-Gi and further signaling assays in CaSR-AA cells, related to Figure 6

(**A**) Ligand-induced BRET ratios measured between CaSR-Nluc and Venus-mGq in cells expressing CaSR-AA pre-treated with vehicle or Dyngo with AUC. N=9. (**B**) Dose-response curve generated from AUC of BRET between Nluc-GRK3 and Venus-Gβγ in cells expressing Gα11 and CaSR-AA and pre-treated with vehicle or Dyngo. N=7. (**C**) Ligand-induced IP_3 biosensor responses in CaSR-AA expressing cells pre-treated with either vehicle or Dyngo. N=4. BRET data shows vehicle subtracted responses with mean+SEM. (**D**) Western blot of p-ERK1/2 responses in cells expressing CaSR-WT or CaSR-AA. Cells were exposed to a five-minute pulse of Ca^{2+}_e, then incubated in basal Ca^{2+}_e (0.1mM) for times indicated. (Below) Densitometry showing p-ERK1/2 normalized to total ERK1/2 from N=4 biological replicates. (**E**) cAMP Glosensor responses for CaSR-WT or CaSR-AA. Data was normalized to 0.1mM Ca^{2+} and forskolin. (**F**) Dose-response generated from AUC in E. N=4. Statistical analyses were performed by unpaired t-test in A, one-way ANOVA with Sidak's test in B, and two-way ANOVA with Sidak's test in F.

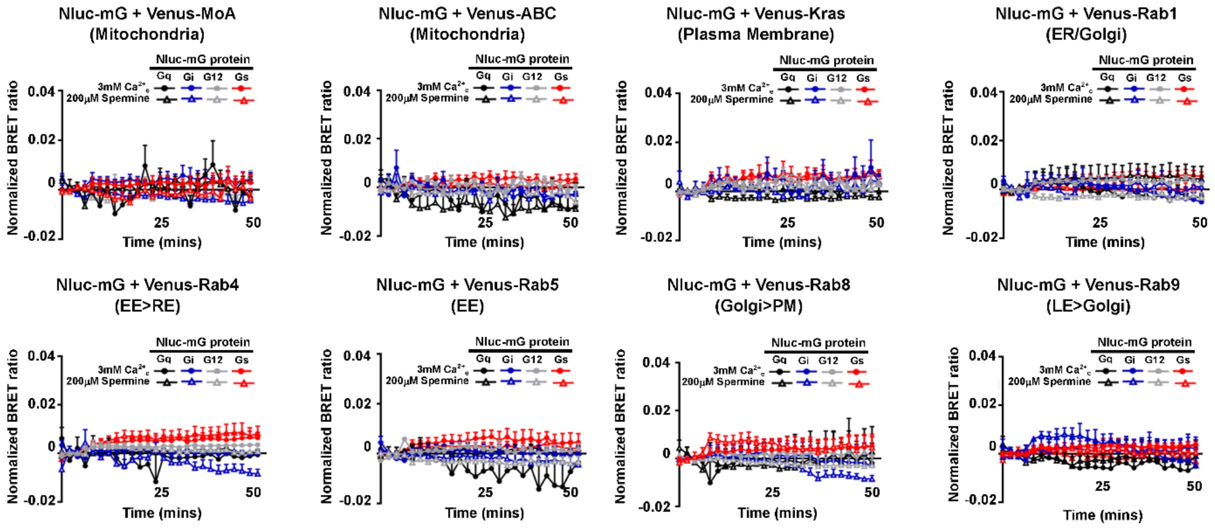

Figure S9 CaSR agonists do not recruit mini-G proteins to intracellular membranes in the absence of receptor, related to Figure 6

Ligand-induced BRET ratios measured between Venus-tagged intracellular markers and Nluc-tagged mini-G proteins in HEK293 cells without CaSR. N=4 for mGq, mGi and mG12, and N=3 for mGs. BRET data shows vehicle subtracted responses with mean+SEM. Statistical analyses comparing AUC showed no significant differences.

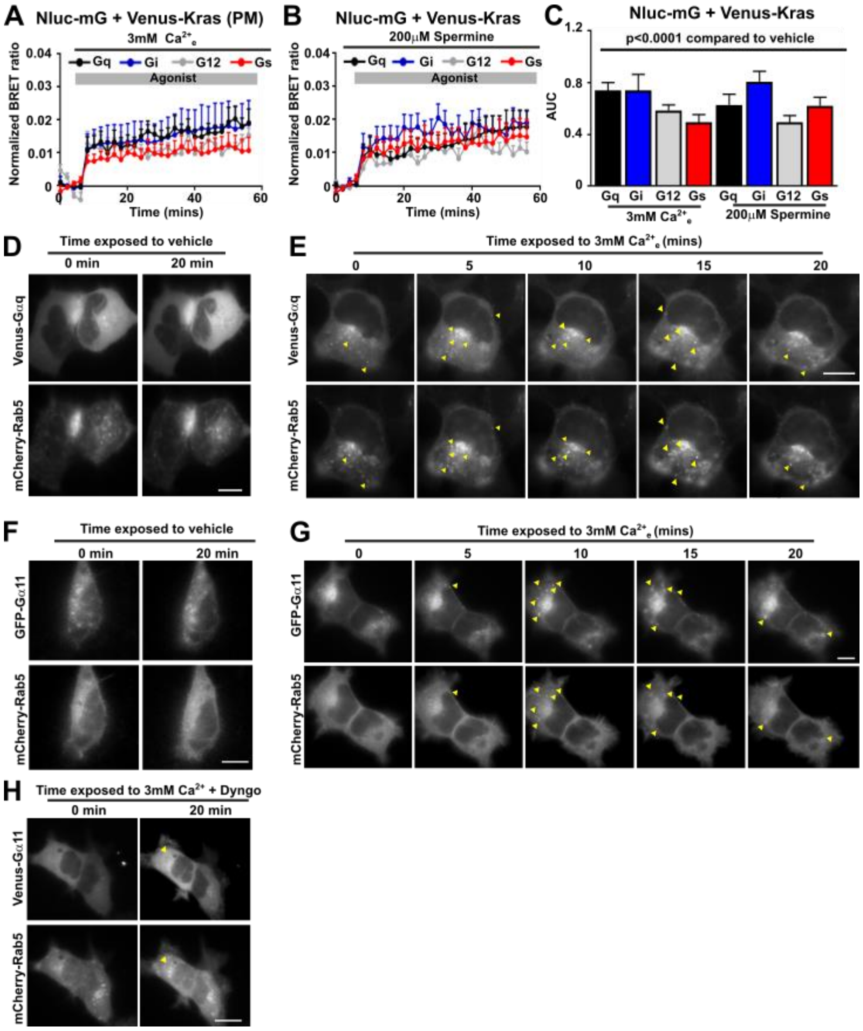

Figure S10 Gαq/11 are recruited to early endosomes following CaSR stimulation, related to Figure 6

(**A**) Ligand-induced BRET ratios measured between Venus-Kras and Nluc-tagged mini-G proteins with (A) 3mM $CaCl_2$ or (B) 100µM spermine, with (**C**) AUC. N=8. (**D-E**) Representative HILO images from HEK-CaSR cells transfected with Venus-mGq and exposed to (D) vehicle (N=15) or (E) 3mM Ca^{2+}_e (N=21) from N=4 biological replicates. (**F-G**) HILO images from HEK-CaSR cells transfected with GFP-Gα11, and mCherry-Rab5, and exposed to (F) vehicle or (G) 3mM Ca^{2+}_e. Co-localized vesicles are highlighted with arrows. N=21 cells (vehicle) and N=25 cells (3mM Ca^{2+}_e) from 5 biological replicates. (**H**) HILO images from HEK-CaSR cells exposed to Dyngo and transfected with mCherry-Rab5 and GFP-Gα11. N=25 cells from 4 biological replicates. BRET data was vehicle subtracted. Scale bar, 5µm. Data were normalized to vehicle and statistical analyses were performed by one-way ANOVA with Sidak's test in C.

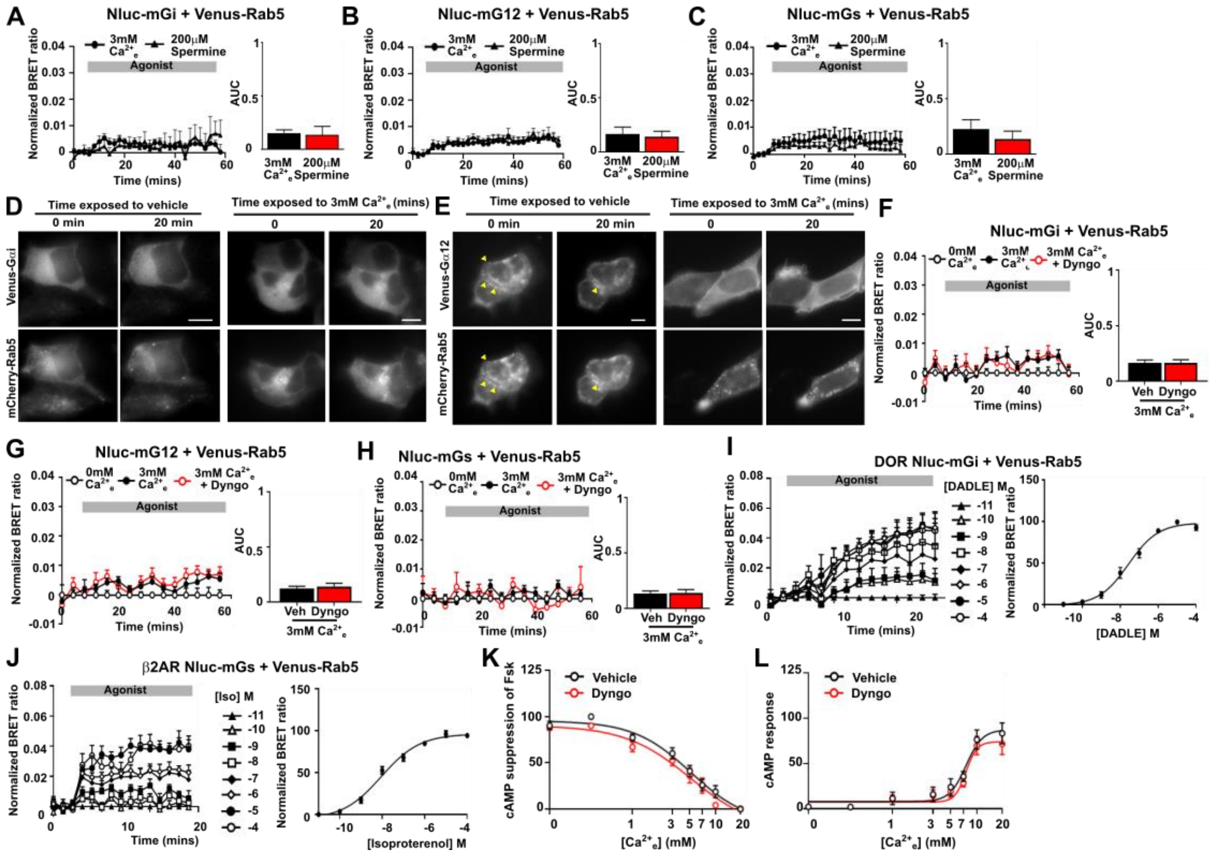

Figure S11 Other G proteins are not recruited to early endosomes following CaSR stimulation, related to Figure 6

BRET between: (**A**) Venus-Rab5 and Nluc-mGi (N=8), (**B**) Venus-Rab5 and Nluc-mG12 (N=13), (**C**) Venus-Rab5 and Nluc-mGs (N=5). (**D**) HILO images from HEK-CaSR cells transfected with mCherry-Rab5 and Venus-mGi and exposed to vehicle (N=15 cells) or 3mM $Ca^{2+}{}_e$ (N=17 cells) from N=3-4 biological replicates (**E**) HILO images from HEK-CaSR cells transfected with mCherry-Rab5 and Venus-mG12 exposed to vehicle or 3mM $Ca^{2+}{}_e$. N=9 from 3 biological replicates for both. (**F-H**) BRET ratios between Venus-Rab5 and (F) Nluc-mGi, (G) Nluc-mG12, (H) Nluc-mGs, with AUC in cells exposed to 0 or 3mM $Ca^{2+}{}_e$ and vehicle or Dyngo-4a. N=5 for all. (**I-J**) Control experiments demonstrating that mGs and mGi can be recruited to Rab5-containing endosomes by other GPCRs. (I) Ligand-induced BRET ratios measured between Nluc-mGi and Venus-Rab5 in cells expressing the δ-opioid receptor (DOR), with (right) dose-response generated from AUC. N=6. (J) Ligand-induced BRET ratios measured between Nluc-mGs and Venus-Rab5 in cells expressing β2AR, with (right) dose-response generated from AUC. N=7. BRET data was vehicle subtracted. Scale bar, 5μm in all images. (**K-L**) cAMP responses measured in cells exposed to vehicle or Dyngo-4a in the presence of (K) forskolin to assess Gi responses and (L) pertussis toxin to assess Gs responses. N=7 in K and L. Data were normalized to vehicle and statistical analyses were performed by one-way ANOVA with Sidak's test in A-C, F-H, two-way ANOVA with Sidak's multiple-comparisons test in K and L.

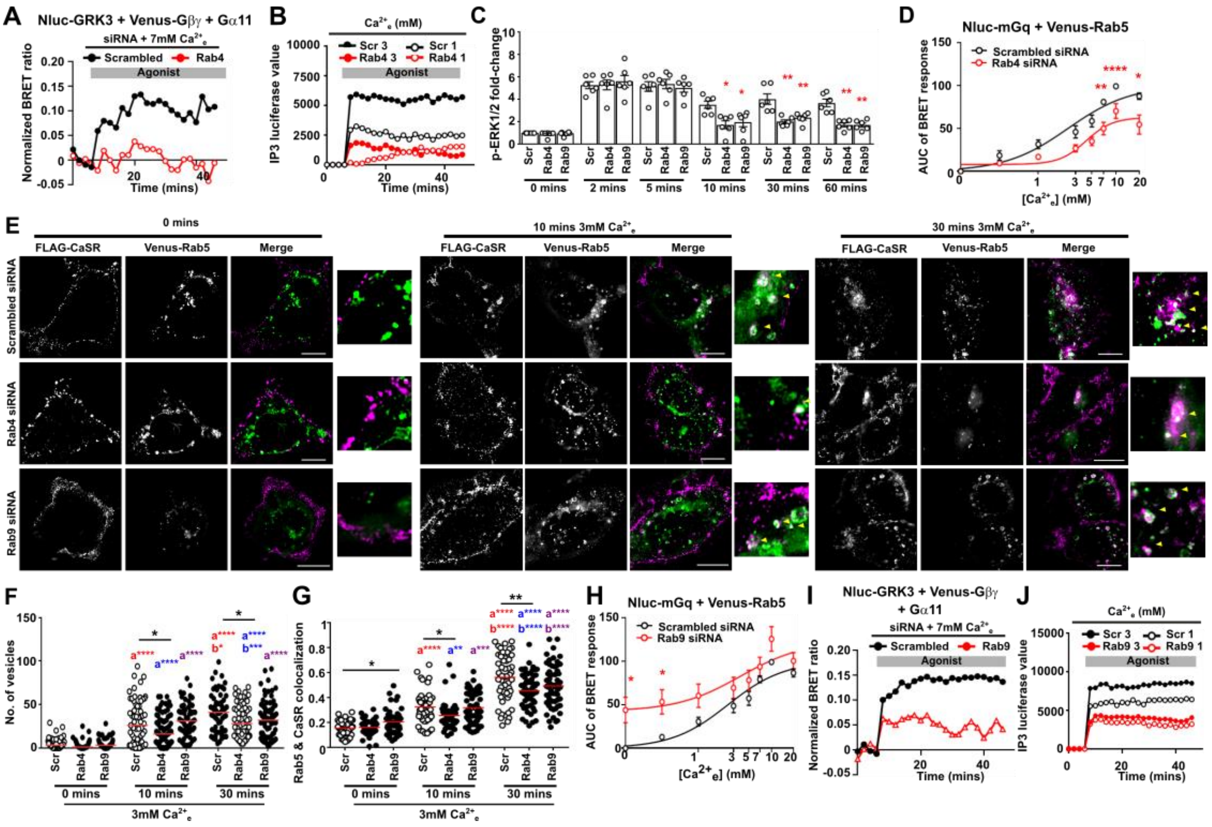

Figure S12 Rab4 and Rab9 siRNA impair CaSR signalling, related to Figure 7 (**A**) Example of ligand-induced BRET responses from Figure 7E between Nluc-GRK3 and Venus-Gβγ in cells expressing Gα11 in HEK-CaSR cells pre-treated with either scrambled or Rab4 siRNA. (**B**) Example of ligand-induced IP3 biosensor responses from Figure 7F in cells pre-treated with either scrambled or Rab4 siRNA. (**C**) pERK1/2 responses in HEK-CaSR cells transfected with scrambled, Rab4 or Rab9 siRNA and exposed to a five-minute pulse of Ca^{2+} measured by AlphaScreen. pERK1/2 responses were normalized to total ERK1/2 responses, then expressed relative to values at 0 minutes as a fold-change. Statistical analyses compare siRNA treatments to scrambled siRNA. N=6. (**D**) BRET between Nluc-mGq and Venus-Rab5 in cells expressing CaSR and transfected with scrambled or Rab4 siRNA (N=6). (**E**) SIM images of cells transfected with FLAG-CaSR and Venus-Rab5 with Scrambled, Rab4 or Rab9 siRNA. Cells were exposed to FLAG antibody and 3mM Ca^{2+}_e for 0, 10 or 30 minutes. Scale, 5µm. Arrows show colocalization. (**F**) Quantification of the total number of vesicles and (**G**) colocalization measured by Pearson's coefficient between FLAG-CaSR and Venus-Rab5. Number of cells from four biological replicates were as follows: Scrambled siRNA 0 mins (51), 10 mins (52), 30 mins (56), Rab4 siRNA 0 mins (38), 10 mins (54), 30 mins (63), Rab9 siRNA 0 mins (50), 10 mins (64), 30 mins (57). (**H**) BRET between Nluc-mGq and Venus-Rab5 in cells expressing CaSR and transfected with scrambled or Rab4 siRNA (N=6). (**I**) Example of BRET from Figure 7L between Nluc-GRK3 and Venus-Gβγ on cells expressing Gα11 in HEK-CaSR cells pre-treated with either scrambled or Rab9 siRNA from Figure 7L. (**J**) Example IP3 biosensor responses in cells pre-treated with either scrambled or Rab9 siRNA from Figure 7M. Statistical analyses were performed by one-way ANOVA with Sidak's multiple-comparisons test in panel C, two-way ANOVA with Sidak's test in D and H. Kruskal-Wallis one-way ANOVA with Dunn's multiple-comparisons test in F and G. Analyses in red compare Scrambled siRNA datasets, in blue the Rab4 siRNA datasets and purple the Rab9 siRNA datasets in F and G. Comparisons with 'a' compare to 0 mins and 'b' to 10 mins.

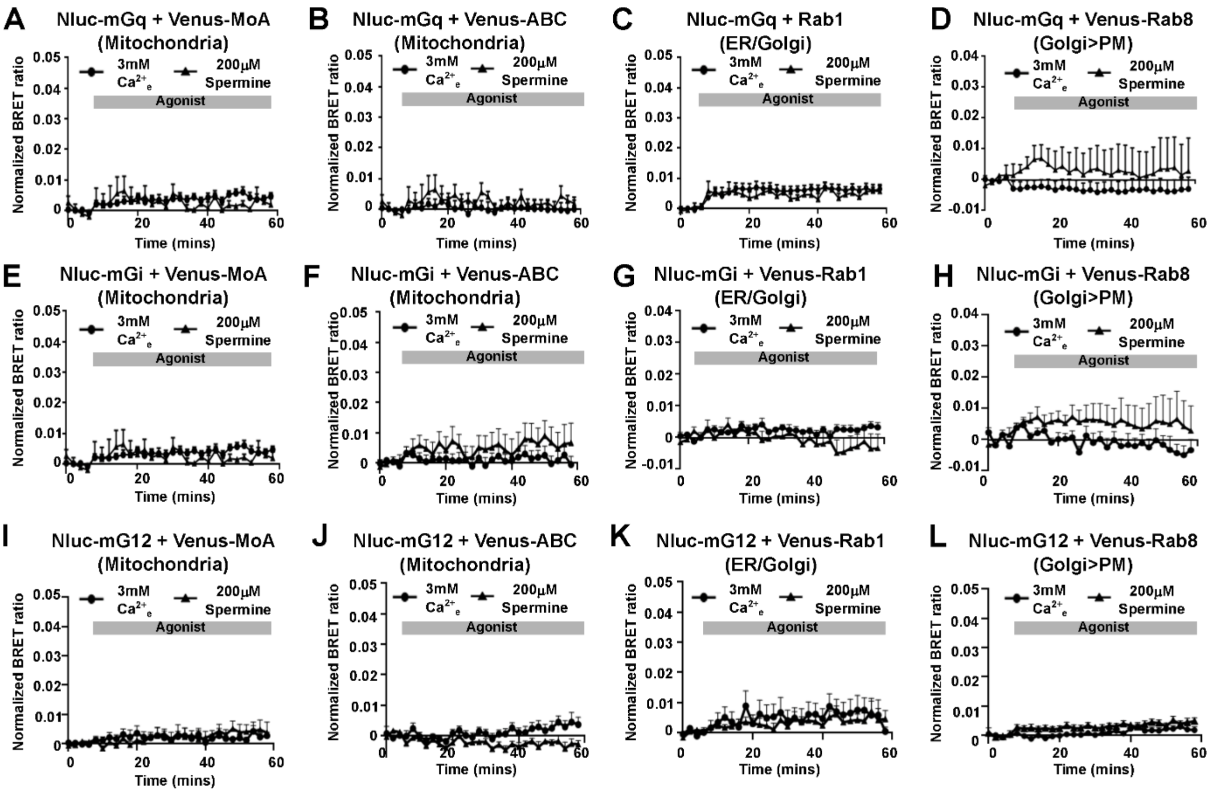

Figure S13 G proteins are not recruited to additional membranes, related to Figure 7

(**A-L**) Ligand-induced BRET ratios between Venus-tagged markers of the mitochondria, ER>Golgi and lysosome and (A-D) Nluc-mGq (N=5-7), (E-H) Nluc-mGi (N=8), (I-L) Nluc-mG12 (N=7-10). BRET data shows vehicle subtracted responses with mean+SEM. Statistical analyses comparing AUC showed no significant differences.

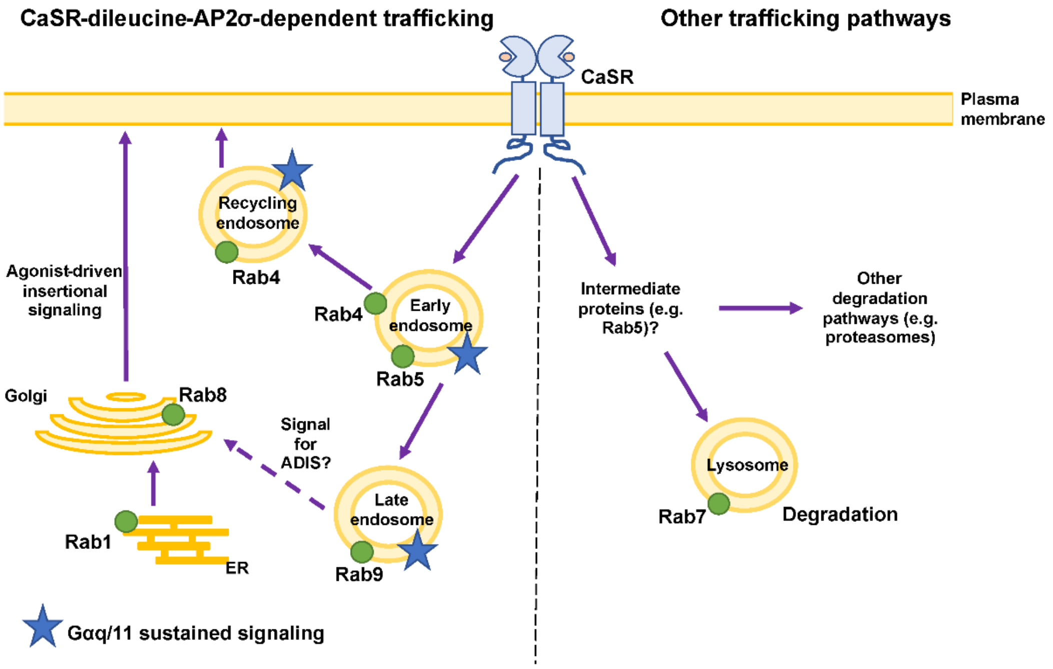

Figure S12 **Summary diagram showing internalization pathways and sites of sustained signaling**

Stimulation of the CaSR directs the receptor to a dynamin-dependent pathway that involves CaSR-dileucine binding to the AP2σ-R15 residue and can elicit Gq signals from intracellular sites (left). In the dileucine-AP2σ pathway, CaSR internalizes to Rab5/Rab4-positive early endosomes, but can then follow different pathways including, trafficking via Rab4 to distinct recycling pathways, or targeting to Rab9-positive late endosomes. In parallel, receptor activation drives forward trafficking of CaSR from the Golgi to the plasma membrane, consistent with the previously described agonist-driven insertional signaling (ADIS) mechanism, which may involve transport from late endosomes to Rab8-positive trans-Golgi vesicles (indicated by hatched line), although the precise mechanism remains to be confirmed. CaSR may traffic from late endosomes to the Golgi, or more likely, sustained signaling communicates with the Golgi to increase ADIS (hatched line) by an unknown mechanism. Stars depict sites of $G\alpha_{q/11}$ signaling at Rab4-, Rab5- and Rab9-positive sites. Activated CaSR can also traffic to other pathways which may or may not require dynamin, the dileucine motif and AP2. CaSR can traffic to Rab7 and proteasome degradation pathways, which may not be agonist-driven. Some constitutive trafficking of CaSR to Rab5-positive endosomes can occur in the absence of the CaSR dileucine motif.

Supplemental References

1. Gao, Y., Robertson, M.J., Rahman, S.N., Seven, A.B., Zhang, C., Meyerowitz, J.G., Panova, O., Hannan, F.M., Thakker, R.V., Brauner-Osborne, H., et al. (2021). Asymmetric activation of the calcium-sensing receptor homodimer. Nature 595, 455-459. 10.1038/s41586-021-03691-0.
